# Disease distribution and temporal trends of salivary gland cancer: A global population‐based study

**DOI:** 10.1002/ctm2.1667

**Published:** 2024-04-29

**Authors:** Junjie Huang, Yat Ching Fung, Wing Sze Pang, Sze Chai Chan, Veeleah Lok, Lin Zhang, Xu Lin, Don Eliseo Lucero‐Prisno, Wanghong Xu, Zhi‐Jie Zheng, Edmar Elcarte, Mellissa Withers, Martin C. S. Wong, NCD Global Health Research Group, Association of Pacific Rim Universities (APRU)

**Affiliations:** ^1^ The Jockey Club School of Public Health and Primary Care, Faculty of Medicine Chinese University of Hong Kong Hong Kong SAR China; ^2^ Centre for Health Education and Health Promotion, Faculty of Medicine The Chinese University of Hong Kong Hong Kong SAR China; ^3^ Department of Global Public Health Karolinska Institute, Karolinska University Hospital Stockholm Sweden; ^4^ Suzhou Industrial Park Monash Research Institute of Science and Technology Suzhou China; ^5^ The School of Public Health and Preventive Medicine Monash University Victoria Australia; ^6^ Department of Thoracic Surgery, The First Affiliated Hospital, School of Medicine Zhejiang University Hangzhou Zhejiang China; ^7^ Department of Global Health and Development London School of Hygiene and Tropical Medicine London UK; ^8^ School of Public Health Fudan University Shanghai China; ^9^ Department of Global Health, School of Public Health Peking University Beijing China; ^10^ College of Nursing University of the Philippines Manila the Philippines; ^11^ Department of Population and Health Sciences Institute for Global Health, University of Southern California Los Angeles California USA

Dear Editor,

Salivary cancer is a rare malignancy worldwide. The number of cases reported is limited and risk factors remain unclear. Some potential risk factors for salivary gland cancer were identified in previous studies, including radiation exposure, smoking, and alcohol drinking.^1^ This study aims to investigate the global disease burden and trends of salivary gland cancer in different countries by age group and sex.

**FIGURE 1:**
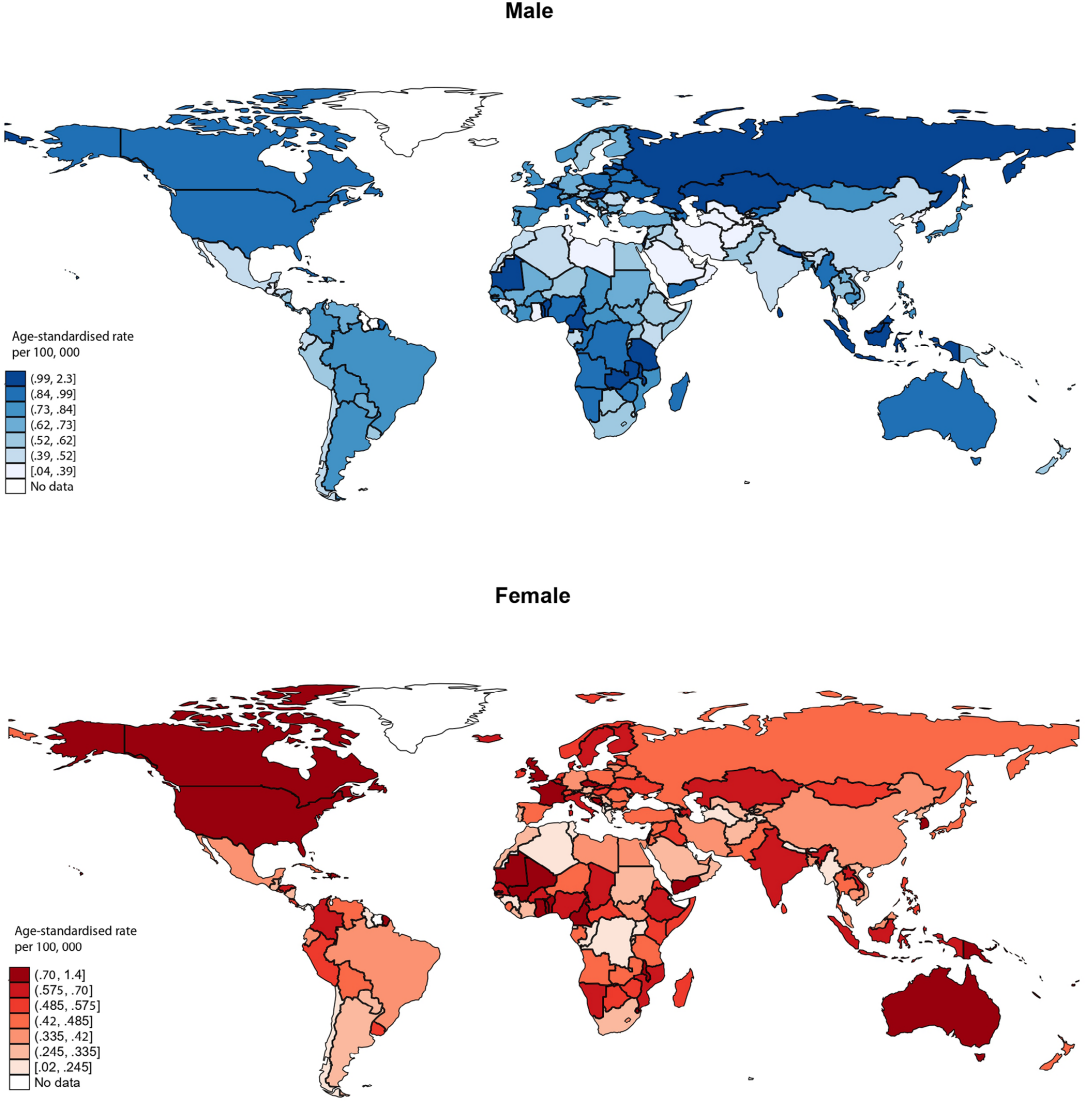
Global incidence of salivary gland cancer incidence by sex.

**FIGURE 2:**
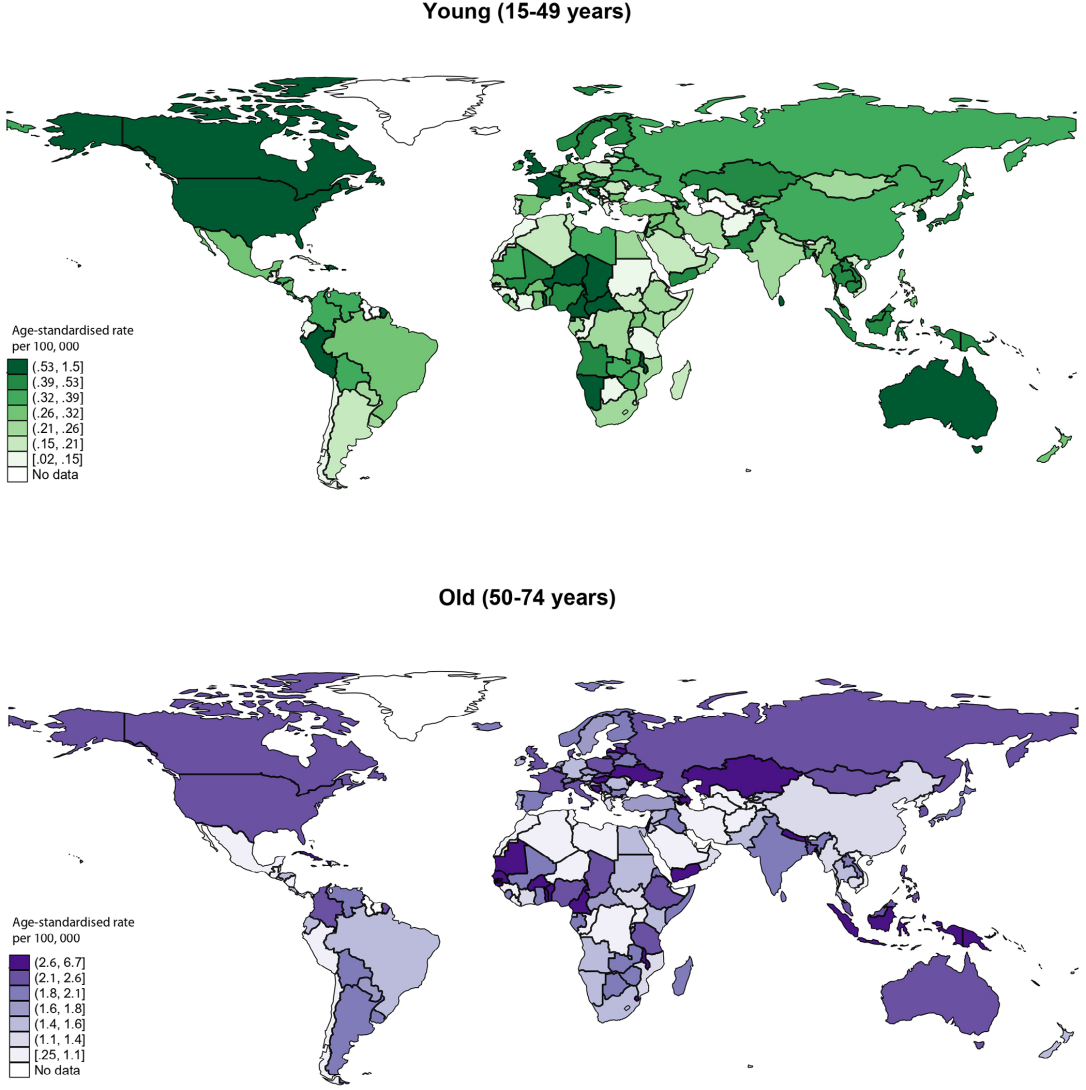
Global incidence of salivary gland cancer incidence by age.

We accessed salivary gland cancer data from the Global Cancer Observatory (GLOBOCAN) and the Cancer Incidence in Five Continents Plus (CI5 Plus).^2^, ^3^ The trend analysis of salivary gland cancer was conducted using joinpoint regression with average annual percentage change (AAPC) with 95% confidence intervals in different age groups (Table S1). The period for incidence and AAPC calculation is the last 10 years of data available for each country, which has been standardised to 2003–2012. We utilised the Human Development Index (HDI) for each country and region from the United Nations to report the disease burden of salivary gland cancer by HDI categories: higher HDI (≥.800) and lower HDI (<.800).^4^

Globally, there were 53 583 new cases of salivary gland cancer with an age‐standardised incidence rate (ASR) of.57 per 100 000 people (Figure 1, 2). Northern America (.85) reported the highest ASR, while the lowest ASR was found in Polynesia (.12). The highest ASRs were observed in regions with very high HDI (.69) and high‐income levels (.7). In terms of age, the ASR of the older population (1.8) was higher than the younger population (.33). In the younger population, region with the highest ASRs was Caribbean (.59). As for the older population, the highest ASRs were observed in Melanesia (2.9) (Tables S2A and S2B).

In countries with higher HDI levels, a diverse trend was observed across all populations, in which Japan (AAPC: 5.90), the United Kingdom (AAPC: 2.69), and Korea (AAPC: 2.48) exhibited the most substantial increases (Figure S1 and Figure S2). Conversely, Lithuania experienced the most significant decline (AAPC: −5.21). No significant trends were observed among males (Figure 3), while females demonstrated a notable rise in countries with higher HDI. Malta (AAPC: 19.61), Martinique (AAPC: 15.62) and Ireland (AAPC: 10.18) experienced the most substantial increases, whereas Poland observed the greatest decrease (AAPC: −9.63). Analysing age subgroups (Figure 4), the most significant increases were reported in Martinique (AAPC: 12.76) and Japan (AAPC: 8.49) in the younger population (aged 15–49) while Switzerland (AAPC: 7.84) ranked the highest in the older population (aged 50+). Cyprus (AAPC: −10.02) and the Philippines (AAPC: −7.30) showed the most significant decrease in younger and older subgroups, respectively.

**FIGURE 3:**
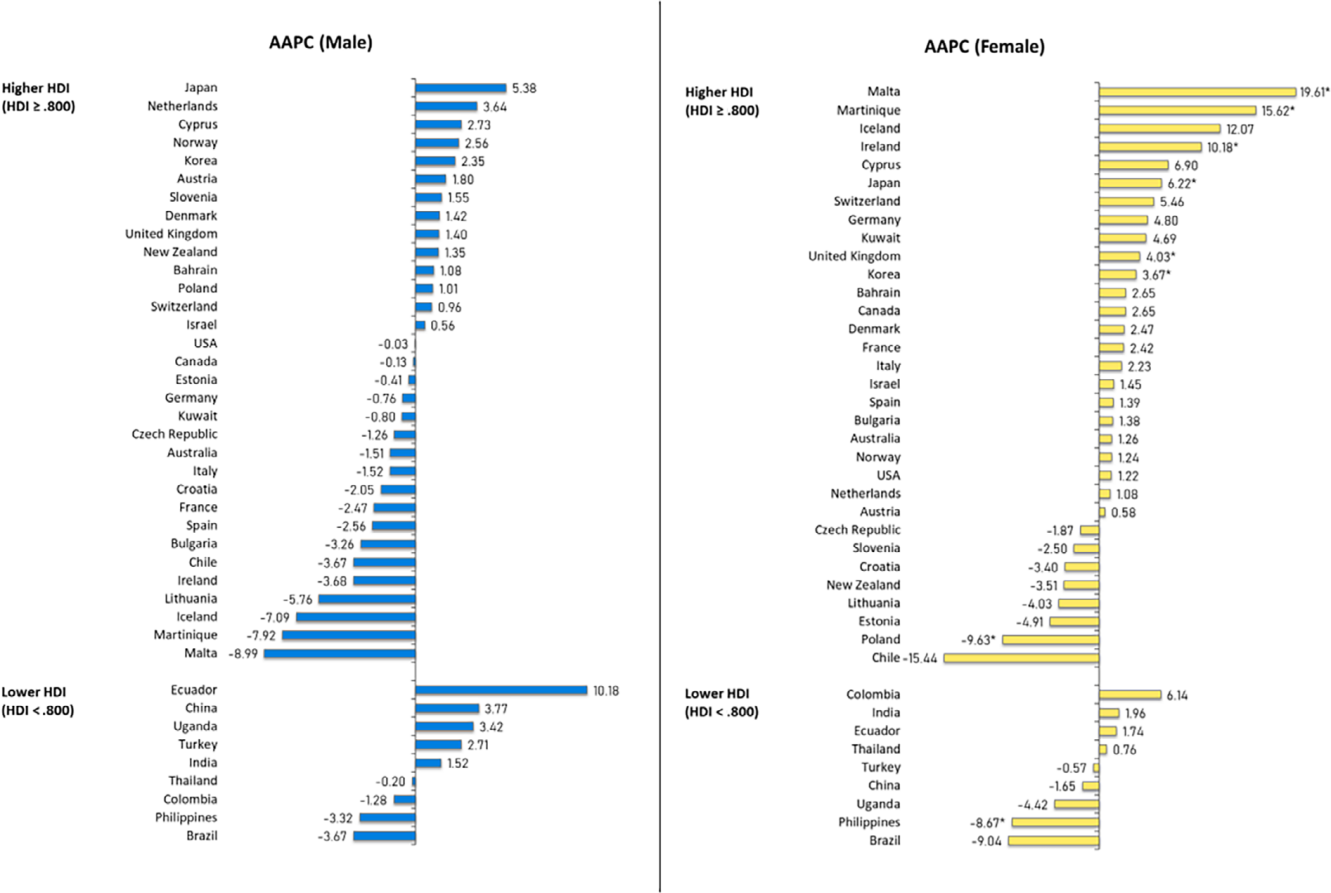
Average annual percentage change (AAPC) of salivary gland cancer incidence by sex, all ages. HDI, Human Development Index.

**FIGURE 4:**
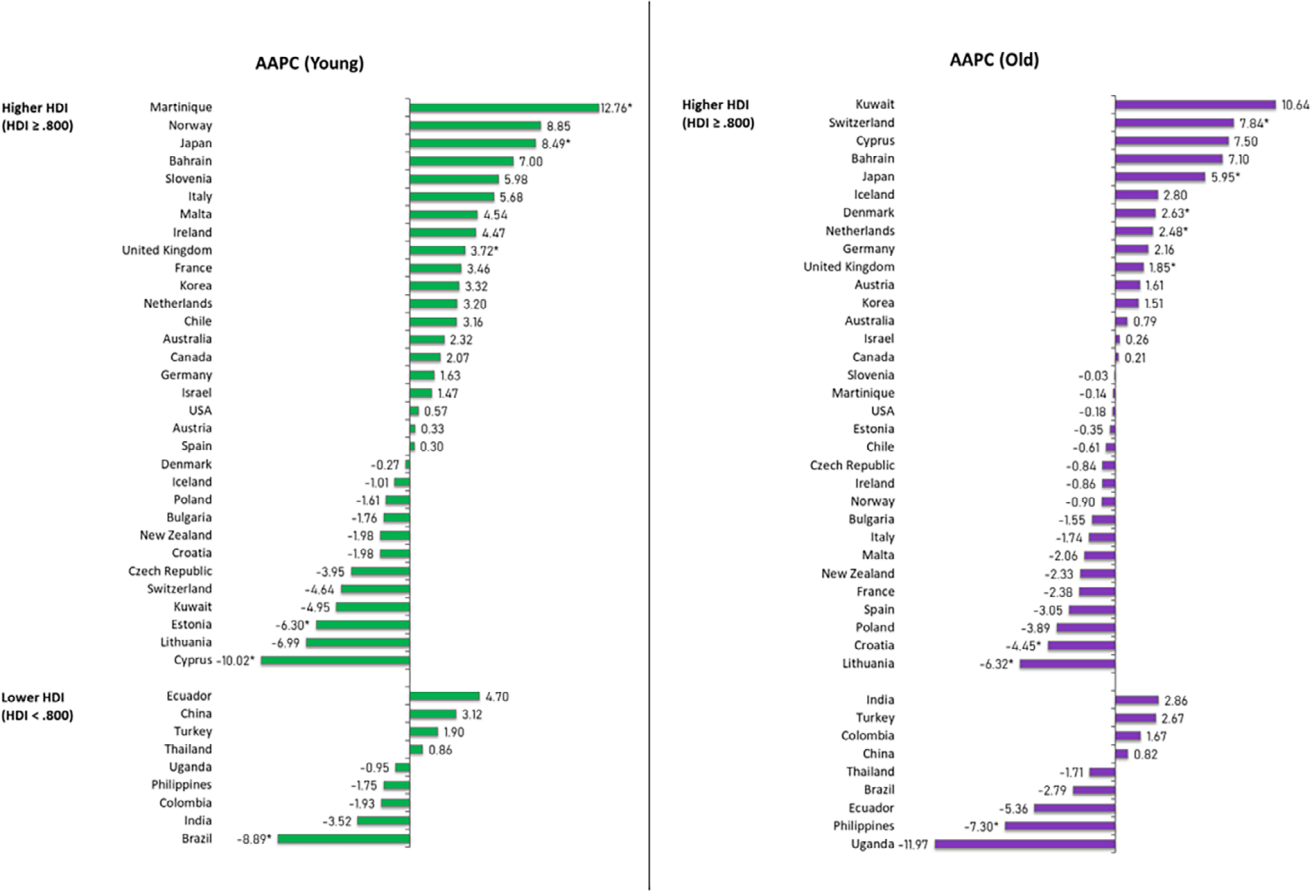
Average annual percentage change (AAPC) of salivary gland cancer incidence by age, both sexes. HDI, Human Development Index.

As for countries with lower HDI levels, the Philippines was the sole significant country, reporting a declining trend throughout the decade across all populations (AAPC: −6.01), females (AAPC: −8.67), and older (AAPC: −7.30) sub‐populations. Additionally, a noteworthy decrease was found among the younger population in Brazil (AAPC: −8.89). No significant trends were reported among the male population. Although decreasing trends were noted in subgroups other than the male population, significance was not observed in other countries.

The study found that the burden of salivary gland cancer was greater in Asian and North European regions with higher HDIs. The burden may be attributed to environmental factors, such as radiation exposure, chemical exposure, occupational exposure, and viral exposure.^5^ Moreover, the disease burden in higher HDI areas may be attributed to the utilisation of local medical services, especially dental services.^6^ The higher incidence of salivary gland cancer in males may be linked to a higher prevalence of smoking and alcohol consumption. Alcohol consumption has been identified to have a positive association with salivary gland cancer, noting that males have higher rates of tobacco and alcohol use than females, with around 30% of men smoking compared to only 20% of women smoking in the United States in 2012.^1^ In addition to lifestyle factors, aging is another potential risk factor for salivary gland cancer. As the body ages, its ability to fight infections and diseases may diminish, rendering it more susceptible to cancer.^7^, ^8^ Age‐related changes in the salivary glands, such as alterations in hormone and saliva composition, may also contribute to the development of salivary gland tumour.^7^, ^8^

The current study highlighted a rising trend of salivary gland cancer among females. As prolonged ovarian activity is related to early menarche and nulliparity, this may result in higher levels of oestradiol and lower sex hormone‐binding globulin levels.^9^ Oestradiol level has been shown related to the risk of breast cancer and other hormone‐dependent cancers as oestradiol may induce cell division, leading to an increased risk of cancer.^10^ It is proposed that a similar mechanism may apply to salivary gland cancer.

The pronounced increase in salivary gland cancer rates in higher‐HDI countries compared to lower‐HDI ones may be attributed to several factors. Firstly, higher‐HDI countries tend to have greater exposure to smoking, alcohol consumption and exposure to certain chemicals or radiation. Additionally, the disparity could be influenced by differences in diagnosis and registry capacity between higher‐ and lower‐HDI countries. Higher‐HDI countries often possess more advanced healthcare infrastructure and resources, including better access to diagnostic tools and more comprehensive cancer registries, leading to higher detection and reporting rates.

However, it is crucial to note the limitations of this study. The cancer data of some low‐ and low–middle‐income countries may be misclassified and under‐reported due to poor infrastructure, inadequate cancer registry coverage and insufficient analytical capacity. Additionally, due to the rarity of salivary gland cancer, there is limited data on the incidence trend of the subtype.

## CONCLUSIONS

In conclusion, the disease burden of salivary gland cancer was significant in both high and low HDI regions, with males and the older population demonstrating a significantly higher incidence. Lifestyle factors such as smoking and alcohol consumption may have contributed to the higher incidence rates observed among males, suggesting that lifestyle modifications may be necessary. Moreover, the current study highlighted a rising trend of salivary gland cancer among females, possibly linked to hormonal factors such as early menarche and nulliparity. Further research is needed to unravel the complex relationship between socioeconomic factors and salivary gland cancer burden, as well as to investigate the potential hormonal mediation of salivary gland cancer.

## AUTHOR CONTRIBUTIONS

Junjie Huang conceptualised, supervised, and drafted the manuscript. Yat Ching Fung performed formal analysis, data curation, and drafted the manuscript. Wing Sze Pang drafted the manuscript. Sze Chai Chan performed formal analysis and data curation. Veeleah Lok, Lin Zhang, Xu Lin, Don Eliseo Lucero‐Prisno III, Wanghong Xu, Zhi‐Jie Zheng, Edmar Elcarte, and Mellissa Withers reviewed and revised the manuscript. Martin C. S. Wong conceptualised, supervised, reviewed, and revised the manuscript.

## CONFLICT OF INTEREST STATEMENT

The authors declare no conflicts of interest.

## ETHICS STATEMENT

This study was approved by the Survey and Behavioural Research Ethics Committee, The Chinese University of Hong Kong (No. SBRE‐20‐332).

## CONSENT TO PARTICIPATE

Not applicable.

## CONSENT FOR PUBLICATION

Not applicable.

## Supporting information

Supporting Information

## Data Availability

The data that support findings of this study are available from the corresponding author, upon reasonable request.
