# Supplementary material for: Disease distribution and temporal trends of salivary gland cancer: A global population‐based study
Source: Clin Transl Med. 2024 Apr 29;14(5):e1667. doi: 10.1002/ctm2.1667 (PMC11058279; doi:10.1002/ctm2.1667)
Supplement: Supplementary file 1 — Supporting Information [file CTM2-14-e1667-s001.docx]

**Supplementary Legends**

**Supplementary Table 1.** Results of Joinpoint regression for trend analysis

**Supplementary Table 2A.** Incidence overall, male, female, younger (15-49), older (50-74)

**Supplementary Table 2B.** Incidence, overall population, Human Development Index, Income level

**Supplementary Figure 1.** Incidence trends for individual countries

**Supplementary Figure 2.** Plots of Joinpoint regression for trend analysis

**Supplementary Table 1.** Results of Joinpoint regression for trend analysis

1. Male

| **Region** | **AAPC** | **Lower CI** | **Upper CI** | **p-value** | **Significant** |
| --- | --- | --- | --- | --- | --- |
| ***Asia*** |  |  |  |  |  |
| Bahrain | 1.08 | -12.19 | 16.34 | 0.865 |  |
| China | 3.77 | -0.40 | 8.11 | 0.071 |  |
| India | 1.52 | -8.68 | 12.85 | 0.751 |  |
| Israel | 0.56 | -3.10 | 4.34 | 0.739 |  |
| Japan | 5.38 | -0.16 | 11.23 | 0.056 |  |
| Korea | 2.35 | -1.13 | 5.96 | 0.189 |  |
| Kuwait | -0.80 | -16.97 | 18.53 | 0.920 |  |
| Philippines | -3.32 | -8.85 | 2.54 | 0.222 |  |
| Thailand | -0.20 | -10.65 | 11.48 | 0.968 |  |
| Turkey | 2.71 | -5.62 | 11.79 | 0.486 |  |
| ***Oceania*** |  |  |  |  |  |
| Australia | -1.51 | -5.24 | 2.36 | 0.439 |  |
| New Zealand | 1.35 | -4.21 | 7.23 | 0.599 |  |
| ***Northern America*** |  |  |  |  |  |
| Canada | -0.13 | -2.34 | 2.13 | 0.897 |  |
| USA | -0.03 | -1.95 | 1.93 | 0.974 |  |
| ***Southern America*** |  |  |  |  |  |
| Brazil | -3.67 | -13.26 | 6.97 | 0.434 |  |
| Chile | -3.67 | -23.38 | 21.11 | 0.749 |  |
| Colombia | -1.28 | -9.31 | 7.46 | 0.735 |  |
| Ecuador | 10.18 | -3.62 | 25.96 | 0.133 |  |
| Martinique | -7.92 | -22.60 | 9.54 | 0.305 |  |
| ***Northern Europe*** |  |  |  |  |  |

| Denmark | 1.42 | -1.41 | 4.33 | 0.284 |
| --- | --- | --- | --- | --- |
| Estonia | -0.41 | -19.34 | 22.98 | 0.966 |
| Iceland | -7.09 | -18.09 | 5.38 | 0.215 |
| Ireland | -3.68 | -9.40 | 2.41 | 0.196 |
| Lithuania | -5.76 | -11.54 | 0.39 | 0.062 |
| Norway | 2.56 | -2.38 | 7.76 | 0.272 |
| United Kingdom | 1.40 | -0.25 | 3.06 | 0.086 |
| ***Western Europe*** |  |  |  |  |
| Austria | 1.80 | -1.92 | 5.66 | 0.300 |
| France | -2.47 | -5.63 | 0.78 | 0.117 |
| Germany | -0.76 | -6.06 | 4.83 | 0.755 |
| Netherlands | 3.64 | -1.10 | 8.61 | 0.116 |
| Switzerland | 0.96 | -3.67 | 5.82 | 0.650 |
| ***Southern Europe*** |  |  |  |  |
| Croatia | -2.05 | -5.61 | 1.64 | 0.233 |
| Cyprus | 2.73 | -7.11 | 13.61 | 0.555 |
| Italy | -1.52 | -12.61 | 10.97 | 0.801 |
| Malta | -8.99 | -20.82 | 4.61 | 0.157 |
| Slovenia | 1.55 | -5.57 | 9.21 | 0.638 |
| Spain | -2.56 | -5.85 | 0.84 | 0.119 |
| ***Eastern Europe*** |  |  |  |  |
| Bulgaria | -3.26 | -6.95 | 0.59 | 0.086 |
| Czech Republic | -1.26 | -4.38 | 1.97 | 0.439 |
| Poland | 1.01 | -9.15 | 12.30 | 0.833 |
| ***Africa*** |  |  |  |  |
| Uganda | 3.42 | -16.13 | 27.52 | 0.721 |

AAPC, annual percentage change; CI, confidence interval; * p values less than 0·05.

1. Female

| **Region** | **AAPC** | **Lower CI** | **Upper CI** | **p-value** | **Significant** |
| --- | --- | --- | --- | --- | --- |
| ***Asia*** |  |  |  |  |  |
| Bahrain | 2.65 | -22.02 | 35.14 | 0.852 |  |
| China | -1.65 | -6.57 | 3.52 | 0.475 |  |
| India | 1.96 | -7.90 | 12.88 | 0.671 |  |
| Israel | 1.45 | -3.58 | 6.76 | 0.532 |  |
| Japan | 6.22 | 1.61 | 11.04 | 0.014 | * |
| Korea | 3.67 | 0.25 | 7.22 | 0.038 | * |
| Kuwait | 4.69 | -4.15 | 14.35 | 0.265 |  |
| Philippines | -8.67 | -12.78 | -4.37 | 0.002 | * |
| Thailand | 0.76 | -7.26 | 9.47 | 0.858 |  |
| Turkey | -0.57 | -10.69 | 10.69 | 0.905 |  |
| ***Oceania*** |  |  |  |  |  |
| Australia | 1.26 | -0.63 | 3.19 | 0.165 |  |
| New Zealand | -3.51 | -9.8 | 3.22 | 0.256 |  |
| ***Northern America*** |  |  |  |  |  |
| Canada | 2.65 | -0.02 | 5.39 | 0.052 |  |
| USA | 1.22 | -1.18 | 3.67 | 0.277 |  |
| ***Southern America*** |  |  |  |  |  |
| Brazil | -9.04 | -19.60 | 2.90 | 0.114 |  |
| Chile | -15.44 | -37.53 | 14.46 | 0.278 |  |
| Colombia | 6.14 | -1.12 | 13.93 | 0.089 |  |
| Ecuador | 1.74 | -15.99 | 23.22 | 0.840 |  |
| Martinique | 15.62 | 0.05 | 33.61 | 0.049 | * |
| ***Northern Europe*** |  |  |  |  |  |
| Denmark | 2.47 | -2.06 | 7.20 | 0.248 |  |

| Estonia | -4.91 | -13.56 | 4.61 | 0.259 |  |
| --- | --- | --- | --- | --- | --- |
| Iceland | 12.07 | -0.83 | 26.65 | 0.064 |  |
| Ireland | 10.18 | 0.80 | 20.42 | 0.036 | * |
| Lithuania | -4.03 | -8.78 | 0.98 | 0.099 |  |
| Norway | 1.24 | -7.34 | 10.62 | 0.756 |  |
| United Kingdom | 4.03 | 2.42 | 5.66 | <0.001 | * |
| ***Western Europe*** |  |  |  |  |  |
| Austria | 0.58 | -3.34 | 4.65 | 0.746 |  |
| France | 2.42 | -2.38 | 7.46 | 0.283 |  |
| Germany | 4.80 | -4.23 | 14.68 | 0.264 |  |
| Netherlands | 1.08 | -1.66 | 3.89 | 0.394 |  |
| Switzerland | 5.46 | -4.64 | 16.63 | 0.258 |  |
| ***Southern Europe*** |  |  |  |  |  |
| Croatia | -3.40 | -9.30 | 2.88 | 0.241 |  |
| Cyprus | 6.90 | -8.58 | 25.00 | 0.354 |  |
| Italy | 2.23 | -4.25 | 9.16 | 0.460 |  |
| Malta | 19.61 | 7.49 | 33.10 | 0.005 | * |
| Slovenia | -2.50 | -10.56 | 6.28 | 0.517 |  |
| Spain | 1.39 | -6.78 | 10.28 | 0.715 |  |
| ***Eastern Europe*** |  |  |  |  |  |
| Bulgaria | 1.38 | -3.74 | 6.78 | 0.559 |  |
| Czech Republic | -1.87 | -6.52 | 3.02 | 0.397 |  |
| Poland | -9.63 | -18.02 | -0.39 | 0.043 | * |
| ***Africa*** |  |  |  |  |  |
| Uganda | -4.42 | -25.01 | 21.82 | 0.679 |  |

AAPC, annual percentage change; CI, confidence interval; * p values less than 0·05.

1. Both

| **Region** | **AAPC** | **Lower CI** | **Upper CI** | **p-value** | **Significant** |
| --- | --- | --- | --- | --- | --- |
| ***Asia*** |  |  |  |  |  |
| Bahrain | 1.42 | -18.96 | 26.92 | 0.889 |  |
| China | 1.49 | -1.80 | 4.90 | 0.330 |  |
| India | 2.43 | -4.49 | 9.85 | 0.451 |  |
| Israel | 0.96 | -1.24 | 3.21 | 0.346 |  |
| Japan | 5.90 | 1.24 | 10.78 | 0.019 | * |
| Korea | 2.48 | 0.40 | 4.61 | 0.025 | * |
| Kuwait | 5.33 | -12.41 | 26.67 | 0.534 |  |
| Philippines | -6.01 | -10.13 | -1.70 | 0.013 | * |
| Thailand | -0.67 | -6.53 | 5.55 | 0.804 |  |
| Turkey | 1.01 | -6.27 | 8.85 | 0.765 |  |
| ***Oceania*** |  |  |  |  |  |
| Australia | -0.21 | -2.94 | 2.59 | 0.881 |  |
| New Zealand | -1.25 | -6.59 | 4.38 | 0.614 |  |
| ***Northern America*** |  |  |  |  |  |
| Canada | 1.09 | -0.71 | 2.93 | 0.202 |  |
| USA | 0.49 | -1.03 | 2.04 | 0.478 |  |
| ***Southern America*** |  |  |  |  |  |
| Brazil | -5.37 | -12.9 | 2.80 | 0.163 |  |
| Chile | -6.15 | -22.08 | 13.04 | 0.454 |  |
| Colombia | 2.10 | -4.21 | 8.83 | 0.474 |  |
| Ecuador | 5.62 | -9.00 | 22.59 | 0.422 |  |
| Martinique | 12.68 | -4.07 | 32.36 | 0.126 |  |
| ***Northern Europe*** |  |  |  |  |  |
| Denmark | 1.87 | -0.83 | 4.64 | 0.151 |  |

| Estonia | -2.94 | -10.38 | 5.12 | 0.414 |  |
| --- | --- | --- | --- | --- | --- |
| Iceland | 7.71 | -4.20 | 21.10 | 0.182 |  |
| Ireland | 1.00 | -0.76 | 2.79 | 0.227 |  |
| Lithuania | -5.21 | -8.94 | -1.32 | 0.015 | * |
| Norway | 2.27 | -1.53 | 6.21 | 0.209 |  |
| United Kingdom | 2.69 | 1.33 | 4.07 | 0.002 | * |
| ***Western Europe*** |  |  |  |  |  |
| Austria | 1.52 | -1.19 | 4.31 | 0.235 |  |
| France | 0.02 | -1.58 | 1.65 | 0.978 |  |
| Germany | 1.29 | -3.78 | 6.64 | 0.580 |  |
| Netherlands | 2.52 | -0.06 | 5.17 | 0.054 |  |
| Switzerland | 2.80 | -2.89 | 8.83 | 0.295 |  |
| ***Southern Europe*** |  |  |  |  |  |
| Croatia | -2.55 | -5.82 | 0.84 | 0.119 |  |
| Cyprus | 1.05 | -6.56 | 9.28 | 0.765 |  |
| Italy | 0.85 | -3.78 | 5.70 | 0.689 |  |
| Malta | 2.43 | -5.16 | 10.63 | 0.492 |  |
| Slovenia | -0.14 | -5.81 | 5.87 | 0.957 |  |
| Spain | -0.94 | -5.12 | 3.41 | 0.625 |  |
| ***Eastern Europe*** |  |  |  |  |  |
| Bulgaria | -1.52 | -4.44 | 1.50 | 0.277 |  |
| Czech Republic | -1.30 | -5.36 | 2.94 | 0.543 |  |
| Poland | -3.57 | -11.76 | 5.39 | 0.373 |  |
| ***Africa*** |  |  |  |  |  |
| Uganda | -1.70 | -17.97 | 17.81 | 0.833 |  |

AAPC, annual percentage change; CI, confidence interval; * p values less than 0·05.

1. Young

| **Region** | **AAPC** | **Lower CI** | **Upper CI** | **p-value** | **Significant** |
| --- | --- | --- | --- | --- | --- |
| ***Asia*** |  |  |  |  |  |
| Bahrain | 7.00 | -15.44 | 35.40 | 0.573 |  |
| China | 3.12 | -1.95 | 8.45 | 0.198 |  |
| India | -3.52 | -19.63 | 15.81 | 0.700 |  |
| Israel | 1.47 | -3.96 | 7.21 | 0.557 |  |
| Japan | 8.49 | 1.37 | 16.10 | 0.024 | * |
| Korea | 3.32 | -0.96 | 7.78 | 0.113 |  |
| Kuwait | -4.95 | -15.51 | 6.94 | 0.350 |  |
| Philippines | -1.75 | -9.20 | 6.32 | 0.620 |  |
| Thailand | 0.86 | -6.81 | 9.16 | 0.809 |  |
| Turkey | 1.90 | -5.19 | 9.52 | 0.564 |  |
| ***Oceania*** |  |  |  |  |  |
| Australia | 2.32 | -0.77 | 5.50 | 0.123 |  |
| New Zealand | -1.98 | -10.65 | 7.54 | 0.632 |  |
| ***Northern America*** |  |  |  |  |  |
| Canada | 2.07 | -0.47 | 4.67 | 0.098 |  |
| USA | 0.57 | -2.13 | 3.34 | 0.644 |  |
| ***Southern America*** |  |  |  |  |  |
| Brazil | -8.89 | -16.42 | -0.69 | 0.037 | * |
| Chile | 3.16 | -8.39 | 16.18 | 0.607 |  |
| Colombia | -1.93 | -12.8 | 10.3 | 0.712 |  |
| Ecuador | 4.70 | -9.15 | 20.66 | 0.477 |  |
| Martinique | 12.76 | 0.62 | 26.37 | 0.041 | * |
| ***Northern Europe*** |  |  |  |  |  |
| Denmark | -0.27 | -9.52 | 9.91 | 0.950 |  |

| Estonia | -6.30 | -10.60 | -1.80 | 0.013 | * |
| --- | --- | --- | --- | --- | --- |
| Iceland | -1.01 | -8.23 | 6.78 | 0.765 |  |
| Ireland | 4.47 | -6.63 | 16.90 | 0.396 |  |
| Lithuania | -6.99 | -16.83 | 4.01 | 0.173 |  |
| Norway | 8.85 | -3.81 | 23.17 | 0.152 |  |
| United Kingdom | 3.72 | 1.06 | 6.45 | 0.012 | * |
| ***Western Europe*** |  |  |  |  |  |
| Austria | 0.33 | -6.97 | 8.19 | 0.923 |  |
| France | 3.46 | -4.34 | 11.89 | 0.346 |  |
| Germany | 1.63 | -11.80 | 17.11 | 0.799 |  |
| Netherlands | 3.20 | -3.47 | 10.34 | 0.308 |  |
| Switzerland | -4.64 | -20.89 | 14.95 | 0.574 |  |
| ***Southern Europe*** |  |  |  |  |  |
| Croatia | -1.98 | -9.21 | 5.82 | 0.563 |  |
| Cyprus | -10.02 | -17.53 | -1.84 | 0.023 | * |
| Italy | 5.68 | -1.15 | 12.98 | 0.093 |  |
| Malta | 4.54 | -1.11 | 10.51 | 0.103 |  |
| Slovenia | 5.98 | -7.53 | 21.48 | 0.355 |  |
| Spain | 0.30 | -7.37 | 8.60 | 0.934 |  |
| ***Eastern Europe*** |  |  |  |  |  |
| Bulgaria | -1.76 | -10.88 | 8.29 | 0.685 |  |
| Czech Republic | -3.95 | -10.03 | 2.54 | 0.193 |  |
| Poland | -1.61 | -14.54 | 13.28 | 0.798 |  |
| ***Africa*** |  |  |  |  |  |
| Uganda | -0.95 | -14.90 | 15.29 | 0.888 |  |

AAPC, annual percentage change; CI, confidence interval; * p values less than 0·05.

1. Old

| **Region** | **AAPC** | **Lower CI** | **Upper CI** | **p-value** | **Significant** |
| --- | --- | --- | --- | --- | --- |
| ***Asia*** |  |  |  |  |  |
| Bahrain | 7.10 | -1.75 | 16.74 | 0.104 |  |
| China | 0.82 | -4.17 | 6.07 | 0.720 |  |
| India | 2.86 | -5.62 | 12.10 | 0.471 |  |
| Israel | 0.26 | -2.95 | 3.58 | 0.858 |  |
| Japan | 5.95 | 2.54 | 9.46 | 0.004 | * |
| Korea | 1.51 | -1.15 | 4.24 | 0.229 |  |
| Kuwait | 10.64 | -2.31 | 25.32 | 0.111 |  |
| Philippines | -7.30 | -13.61 | -0.53 | 0.038 | * |
| Thailand | -1.71 | -11.77 | 9.49 | 0.722 |  |
| Turkey | 2.67 | -5.49 | 11.54 | 0.484 |  |
| ***Oceania*** |  |  |  |  |  |
| Australia | 0.79 | -1.68 | 3.31 | 0.486 |  |
| New Zealand | -2.33 | -7.05 | 2.62 | 0.303 |  |
| ***Northern America*** |  |  |  |  |  |
| Canada | 0.21 | -2.53 | 3.03 | 0.865 |  |
| USA | -0.18 | -1.87 | 1.53 | 0.810 |  |
| ***Southern America*** |  |  |  |  |  |
| Brazil | -2.79 | -11.10 | 6.30 | 0.486 |  |
| Chile | -0.61 | -11.83 | 12.05 | 0.910 |  |
| Colombia | 1.67 | -7.06 | 11.23 | 0.682 |  |
| Ecuador | -5.36 | -20.80 | 13.08 | 0.496 |  |
| Martinique | -0.14 | -15.97 | 18.66 | 0.985 |  |
| ***Northern Europe*** |  |  |  |  |  |
| Denmark | 2.63 | 0.82 | 4.47 | 0.010 | * |

| Estonia | -0.35 | -10.94 | 11.51 | 0.945 |  |
| --- | --- | --- | --- | --- | --- |
| Iceland | 2.80 | -7.32 | 14.02 | 0.556 |  |
| Ireland | -0.86 | -5.20 | 3.67 | 0.666 |  |
| Lithuania | -6.32 | -10.47 | -1.97 | 0.011 | * |
| Norway | -0.90 | -7.53 | 6.20 | 0.770 |  |
| United Kingdom | 1.85 | 0.12 | 3.61 | 0.039 | * |
| ***Western Europe*** |  |  |  |  |  |
| Austria | 1.61 | -2.38 | 5.77 | 0.384 |  |
| France | -2.38 | -5.86 | 1.24 | 0.165 |  |
| Germany | 2.16 | -2.66 | 7.22 | 0.338 |  |
| Netherlands | 2.48 | 0.78 | 4.21 | 0.010 | * |
| Switzerland | 7.84 | 3.95 | 11.88 | 0.001 | * |
| ***Southern Europe*** |  |  |  |  |  |
| Croatia | -4.45 | -7.96 | -0.81 | 0.023 | * |
| Cyprus | 7.50 | -0.71 | 16.39 | 0.069 |  |
| Italy | -1.74 | -9.25 | 6.39 | 0.624 |  |
| Malta | -2.06 | -13.31 | 10.66 | 0.705 |  |
| Slovenia | -0.03 | -6.57 | 6.96 | 0.991 |  |
| Spain | -3.05 | -9.48 | 3.85 | 0.329 |  |
| ***Eastern Europe*** |  |  |  |  |  |
| Bulgaria | -1.55 | -4.57 | 1.56 | 0.280 |  |
| Czech Republic | -0.84 | -5.17 | 3.69 | 0.712 |  |
| Poland | -3.89 | -11.28 | 4.11 | 0.286 |  |
| ***Africa*** |  |  |  |  |  |
| Uganda | -11.97 | -23.97 | 1.92 | 0.080 |  |

AAPC, annual percentage change; CI, confidence interval; * p values less than 0·05.

| **Supplementary Table 2A. Incidence overall, male, female, younger (15-49), older (50-74)** | | | | | | | | | | |  |  |
| --- | --- | --- | --- | --- | --- | --- | --- | --- | --- | --- | --- | --- |
|  | | | | | | | | | | |  |  |
|  | All Population | | By Sex | | | | | | By Age | | | |
|  |  |  | Male | | | Female | | | Young (15-49) | | Old (50-74) | |
| **Population** | Number | ASR | Number | | ASR | Number | | ASR | Number | ASR | Number | ASR (World) |
| **World** | 53,583 | 0.57 | 29,694 | 0.66 | | 23,889 | 0.50 | | 13,387 | 0.33 | 28,657 | 1.8 |
| **Eastern Asia** | 11,568 | 0.47 | 6,563 | 0.53 | | 5,005 | 0.41 | | 3,314 | 0.36 | 6,489 | 1.3 |
| **South-Central Asia** | 10,665 | 0.56 | 5,060 | 0.53 | | 5,605 | 0.59 | | 2,907 | 0.27 | 6,129 | 1.9 |
| **Northern America** | 5,524 | 0.85 | 3,231 | 0.98 | | 2,293 | 0.75 | | 965 | 0.54 | 2,724 | 2.4 |
| **South-Eastern Asia** | 4,842 | 0.68 | 3,011 | 0.89 | | 1,831 | 0.49 | | 1,349 | 0.37 | 2,883 | 2.3 |
| **Central and Eastern Europe** | 3,539 | 0.67 | 2,057 | 0.95 | | 1,482 | 0.48 | | 542 | 0.33 | 2,173 | 2.4 |
| **South America** | 3,025 | 0.55 | 1,754 | 0.71 | | 1,271 | 0.42 | | 709 | 0.30 | 1,477 | 1.6 |
| **Western Europe** | 2,750 | 0.67 | 1,639 | 0.80 | | 1,111 | 0.56 | | 389 | 0.42 | 1,282 | 1.9 |
| **Southern Europe** | 2,168 | 0.62 | 1,306 | 0.78 | | 862 | 0.48 | | 241 | 0.31 | 981 | 1.9 |
| **Western Africa** | 1,645 | 0.71 | 828 | 0.75 | | 817 | 0.67 | | 642 | 0.41 | 846 | 2.4 |
| **Eastern Africa** | 1,464 | 0.59 | 737 | 0.69 | | 727 | 0.52 | | 472 | 0.24 | 681 | 1.9 |
| **Northern Europe** | 1,460 | 0.74 | 779 | 0.75 | | 681 | 0.74 | | 260 | 0.51 | 674 | 2.0 |
| **Western Asia** | 1,391 | 0.54 | 728 | 0.59 | | 663 | 0.50 | | 424 | 0.27 | 725 | 1.8 |
| **Northern Africa** | 852 | 0.39 | 482 | 0.46 | | 370 | 0.33 | | 238 | 0.19 | 432 | 1.2 |
| **Central America** | 790 | 0.41 | 399 | 0.45 | | 391 | 0.38 | | 282 | 0.29 | 333 | 1.1 |
| **Middle Africa** | 704 | 0.69 | 456 | 1.0 | | 248 | 0.42 | | 325 | 0.47 | 237 | 1.6 |
| **Caribbean** | 452 | 0.80 | 255 | 0.96 | | 197 | 0.68 | | 136 | 0.59 | 214 | 2.2 |
| **Australia and New Zealand** | 417 | 0.81 | 231 | 0.86 | | 186 | 0.78 | | 84 | 0.55 | 202 | 2.3 |
| **Southern Africa** | 255 | 0.43 | 151 | 0.61 | | 104 | 0.31 | | 82 | 0.23 | 133 | 1.4 |
| **Melanesia** | 71 | 0.82 | 27 | 0.69 | | 44 | 0.96 | | 26 | 0.48 | 41 | 2.9 |
| **Polynesia** | 1 | 0.12 | 0 | N/A | | 1 | 0.24 | | 0 | N/A | 1 | 0.66 |

| **Supplementary Table 2B. Incidence, overall population, Human Development Index, Income level** | | | | | | | |
| --- | --- | --- | --- | --- | --- | --- | --- |
|  | | | | | | | |
|  | All Population | |  |  |  |  |  |
| Population | Number | ASR |  |  |  |  |  |
| Very high HDI | 19,391 | 0.69 |  |  |  |  |  |
| High HDI | 18,634 | 0.50 |  |  |  |  |  |
| Medium HDI | 11,839 | 0.55 |  |  |  |  |  |
| Low HDI | 3,706 | 0.66 |  |  |  |  |  |
| High income | 16,297 | 0.70 |  |  |  |  |  |
| Upper middle income | 19,804 | 0.51 |  |  |  |  |  |
| Low middle income | 15,358 | 0.57 |  |  |  |  |  |
| Low income | 2,111 | 0.57 |  |  |  |  |  |
|  |  |  |  |  |  |  |  |
| *HDI: Human Development Index | | |  |  |  |  |  |

**Supplementary Figure 1.** Incidence trends for individual countries

Bahrain: incidence

Age-standard (World) incidence per 100 000

# Asia

## China: Incidence

Age-standard (World) incidence per 100 000

India: Incidence

Age-standard (World) incidence per 100 000

4

3

2

1

0

2003 2006 2009 2012

Male Female

Both Young (15-49)

Old (50-74)

Age-standard (World) incidence per 100 000

2

1.5

1

0.5

0

2003 2006 2009 2012

Male Female

Both Young (15-49)

Old (50-74)

3

2.5

2

1.5

1

0.5

0

2003 2006 2009 2012

Male Female

Both Young (15-49)

Old (50-74)

## Israel: Incidence

Age-standard (World) incidence per 100 000

Japan: Incidence

## Korea: Incidence

4

3

2

1

0

2003 2006 2009 2012

Male Female

Both Young (15-49)

Old (50-74)

2

1.5

1

0.5

0

2003 2006 2009 2012

Male Female

Both Young (15-49)

Old (50-74)

2

1.5

1

0.5

0

2003 2006 2009 2012

Age-standard (World) incidence per 100 000

Male Female

Both Young (15-49)

Old (50-74)

## Kuwait: Incidence

Age-standard (World) incidence per 100 000

Philippines: Incidence

## Thailand: Incidence

2

1.5

1

0.5

0

2003 2006 2009 2012

4

3

|  |
| --- |
|  |
|  |
|  |

2

1

0

2003 2006 2009 2012

2

1.5

1

0.5

0

2003 2006 2009 2012

Age-standard (World) incidence per 100 000

Male Female

Both Young (15-49)

Old (50-74)

Male Female

Both Young (15-49)

Old (50-74)

Male Female

Both Young (15-49） Old (50-74)

## Turkey: Incidence

Age-standard (World) incidence per 100 000

Age-standard (World) incidence per 100 000

3

|  |
| --- |
|  |
|  |

2

1

0

2003 2006 2009 2012

Male Female

Both Young (15-49)

Old (50-74)

# Oceania

## Australia: Incidence New Zealand: Incidence

Age-standard (World) incidence per 100 000

Age-standard (World) incidence per 100 000

3

|  |
| --- |
|  |
|  |

2

1

0

2003 2006 2009 2012

Male Female

Both Young (15-49)

Old (50-74)

2.5

2

1.5

1

0.5

0

2003 2006 2009 2012

Male Female

Both Young (15-49)

Old (50-74)

# Northern America

## Canada: Incidence USA: Incidence

Age-standard (World) incidence per 100 000

Age-standard (World) incidence per 100 000

3 3

|  |
| --- |
|  |
|  |

|  |
| --- |
|  |
|  |

2 2

1 1

0

2003 2006 2009 2012

Male Female

Both Young (15-49)

Old (50-74)

0

2003 2006 2009 2012

Male Female

Both Young (15-49)

Old (50-74)

Brazil: Incidence

Age-standard (World) incidence per 100 000

# Southern America

## Chile: Incidence

Age-standard (World) incidence per 100 000

Colombia: Incidence

Age-standard (World) incidence per 100 000

4

3

2

1

0

2003 2006 2009 2012

Male Female

Both Young (15-49)

Old (50-74)

5

4

3

2

1

0

2003 2006 2009 2012

Male Female

Both Young (15-49)

Old (50-74)

5

4

3

2

1

0

2003 2006 2009 2012

Male Female

Both Young (15-49) Old (50-74)

## Ecuador: Incidence Martinique: Incidence

Age-standard (World) incidence per 100 000

Age-standard (World) incidence per 100 000

2.5

2

1.5

1

0.5

0

2003 2006 2009 2012

Male Female

Both Young (15-49)

Old (50-74)

5

4

3

2

1

0

2003 2006 2009 2012

Male Female

Both Young (15-49)

Old (50-74)

## Denmark: Incidence

Age-standard (World) incidence per 100 000

3

|  |
| --- |
|  |
|  |

2

1

0

2003 2006 2009 2012

Male Female

Both Young (15-49)

Old (50-74)

# Northern Europe

## Estonia: Incidence

Age-standard (World) incidence per 100 000

6

|  |
| --- |
|  |
|  |

4

2

0

2003 2006 2009 2012

Male Female

Both Young (15-49)

Old (50-74)

## Iceland: Incidence

4

Age-standard (World) incidence per 100 000

3

2

1

0

2003 2006 2009 2012

Male Female

Both Young (15-49) Old (50-74)

## Ireland: Incidence

Age-standard (World) incidence per 100 000

3

|  |
| --- |
|  |
|  |

2

1

0

2003 2006 2009 2012

Male Female

Both Young (15-49)

Old (50-74)

## Lithuania: Incidence

4

Age-standard (World) incidence per 100 000

3

2

1

0

2003 2006 2009 2012

Male Female

Both Young (15-49)

Old (50-74)

## Norway: Incidence

3

Age-standard (World) incidence per 100 000

|  |
| --- |
|  |
|  |

2

1

0

2003 2006 2009 2012

Male Female

Both Young (15-49)

Old (50-74)

## United Kingdom: Incidence

Age-standard (World) incidence per 100 000

2.5

2

1.5

1

0.5

0

2003 2006 2009 2012

Male Female

Both Young (15-49)

Old (50-74)

# Western Europe

Age-standard (World) incidence per 100 000

Age-standard (World) incidence per 100 000

## Austria: Incidence

Age-standard (World) incidence per 100 000

France: Incidence

## Germany: Incidence

2.5

2

1.5

1

0.5

0

2003 2006 2009 2012

Male Female

Both Young (15-49)

Old (50-74)

2.5

2

1.5

1

0.5

0

2003 2006 2009 2012

Male Female

Both Young (15-49) Old (50-74)

3

2

|  |
| --- |
|  |
|  |

1

0

2003 2006 2009 2012

Male Female

Both Young (15-49) Old (50-74)

## Netherlands: Incidence Switzerland: Incidence

Age-standard (World) incidence per 100 000

Age-standard (World) incidence per 100 000

2

1.5

1

0.5

0

2003 2006 2009 2012

Male Female

Both Young (15-49)

Old (50-74)

3

2

|  |
| --- |
|  |
|  |

1

0

2003 2006 2009 2012

Male Female

Both Young (15-49)

Old (50-74)

Croatia: Incidence

Age-standard (World) incidence per 100 000

# Southern Europe

## Cyprus: Incidence

Age-standard (World) incidence per 100 000

Italy: Incidence

Age-standard (World) incidence per 100 000

4

3

2

1

0

2003 2006 2009 2012

Male Female

Both Young (15-49)

Old (50-74)

2.5

2

1.5

1

0.5

0

2003 2006 2009 2012

Male Female

Both Young (15-49)

Old (50-74)

3

2

|  |
| --- |
|  |
|  |

1

0

2003 2006 2009 2012

Male Female

Both Young (15-49) Old (50-74）

Age-standard (World) incidence per 100 000

## Malta: Incidence

Age-standard (World) incidence per 100 000

Slovenia: Incidence

## Spain: Incidence

4

3

2

1

0

2003 2006 2009 2012

2.5

2

1.5

1

0.5

0

2003 2006 2009 2012

2.5

2

1.5

1

0.5

0

2003 2006 2009 2012

Age-standard (World) incidence per 100 000

Male Female

Both Young (15-49)

Old (50-74)

Male Female

Both Young (15-49)

Old (50-74)

Male Female

Both Young (15-49)

Old (50-74)

Bulgaria: Incidence

Age-standard (World) incidence per 100 000

# Eastern Europe

## Czech Republic: Incidence

Age-standard (World) incidence per 100 000

Poland: Incidence

Age-standard (World) incidence per 100 000

3

2.5

2

1.5

1

0.5

0

2003 2006 2009 2012

Male Female

Both Young (15-49)

Old (50-74)

3

2.5

2

1.5

1

0.5

0

2003 2006 2009 2012

Male Female

Both Young (15-49)

Old (50-74)

4

3

2

1

0

2003 2006 2009 2012

Male Female

Both Young (15-49)

Old (50-74)

# Africa

## Uganda: Incidence

Age-standard (World) incidence per 100 000

6

5

4

3

2

1

0

2003 2006 2009 2012

Male Female

Both Young (15-49)

Old (50-74)


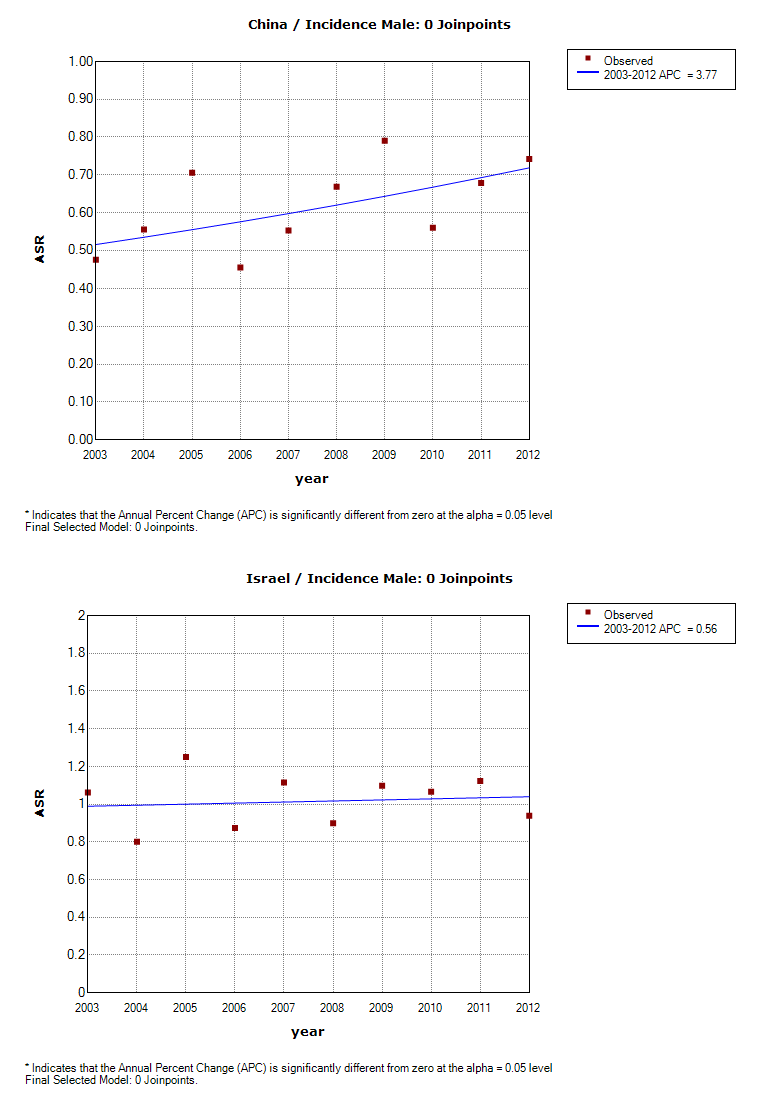

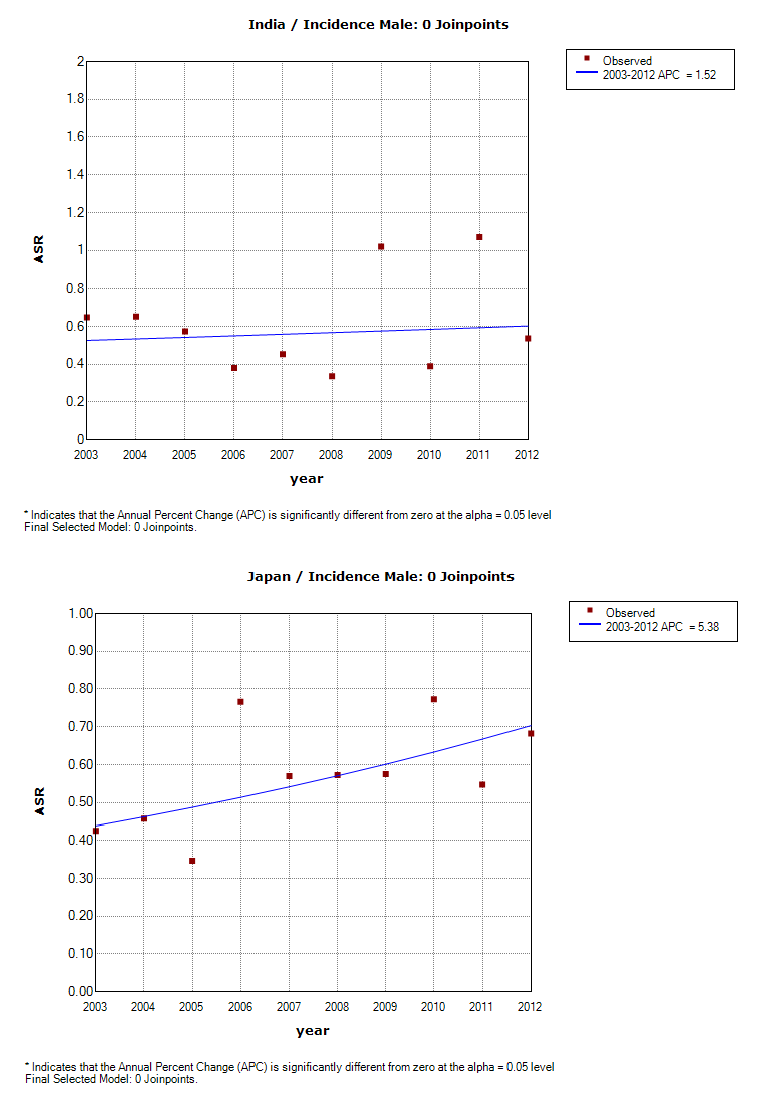
**Supplementary Figure 2.** Plots of Joinpoint regression for trend analysis a.) Male

| **Asia** | |
| --- | --- |
| 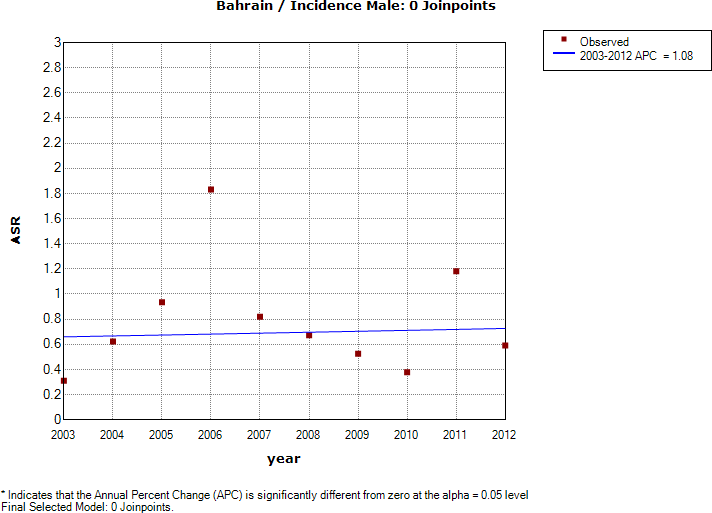 |  |
|  |  |
|  | 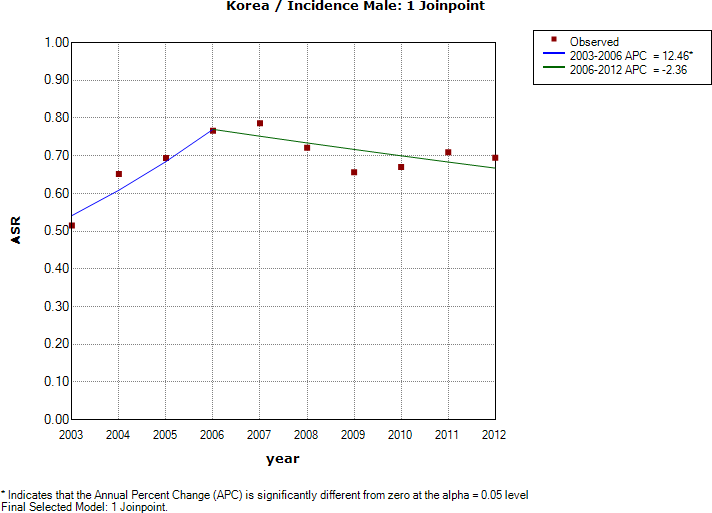 |

| 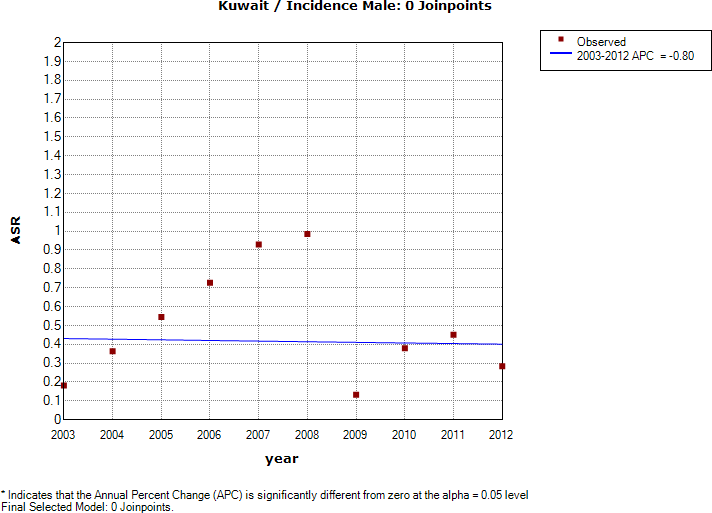 | 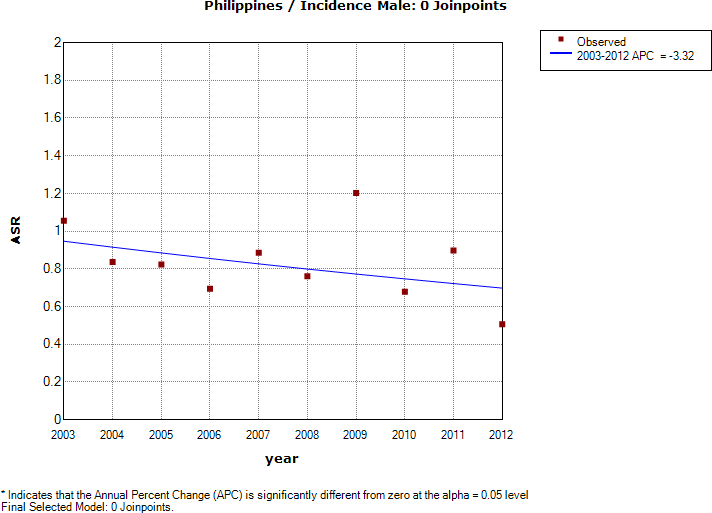 |
| --- | --- |
| 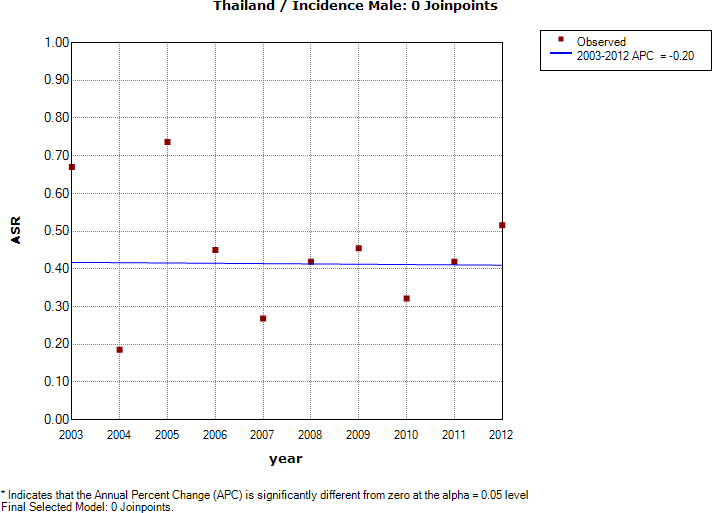 | 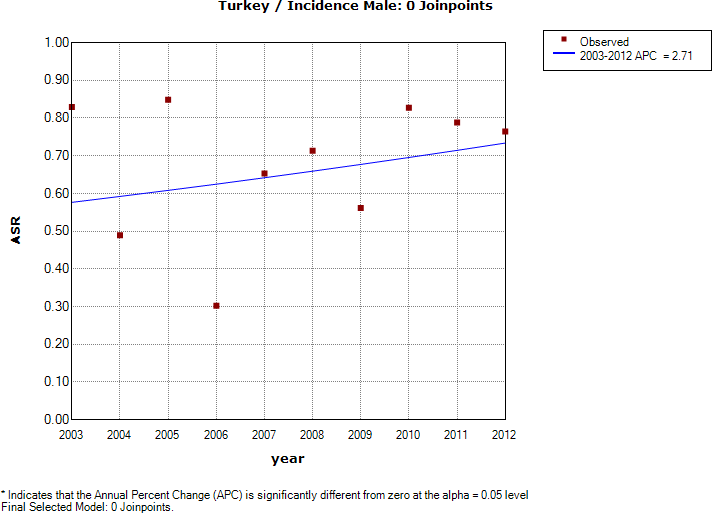 |
| **Oceania** | |
| 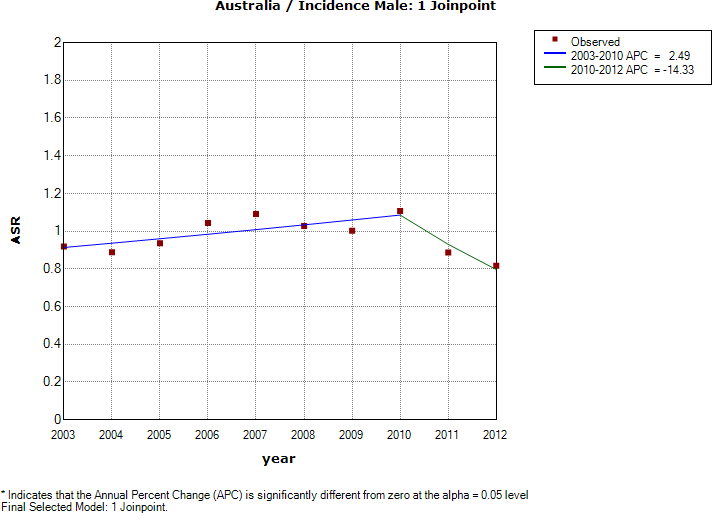 | 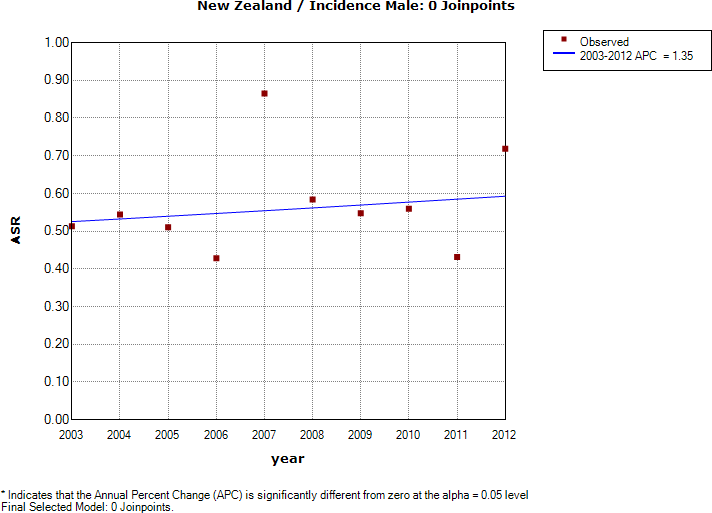 |

| **Northern America** | |
| --- | --- |
| 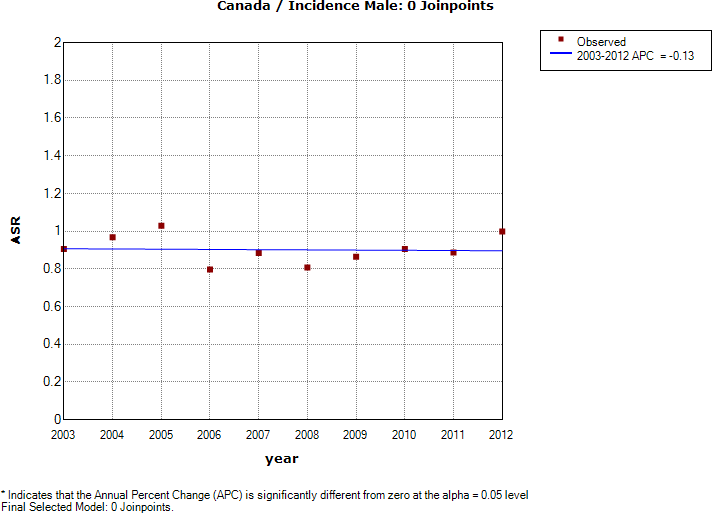 | 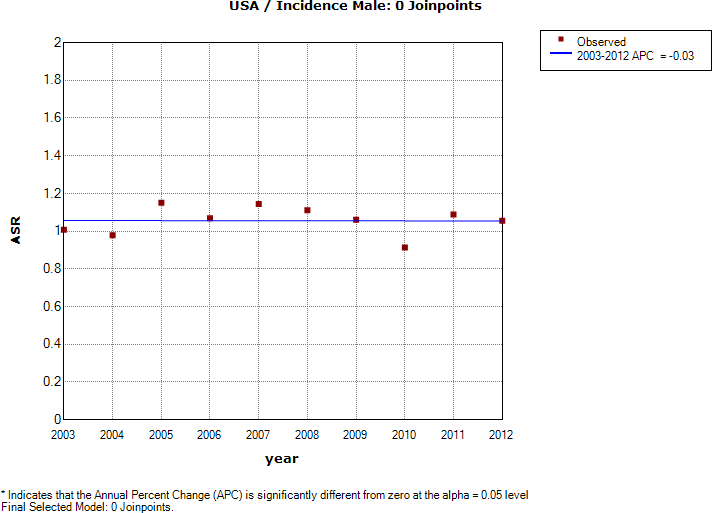 |
| **Southern America** | |
|  |  |
|  |  |
|  |  |


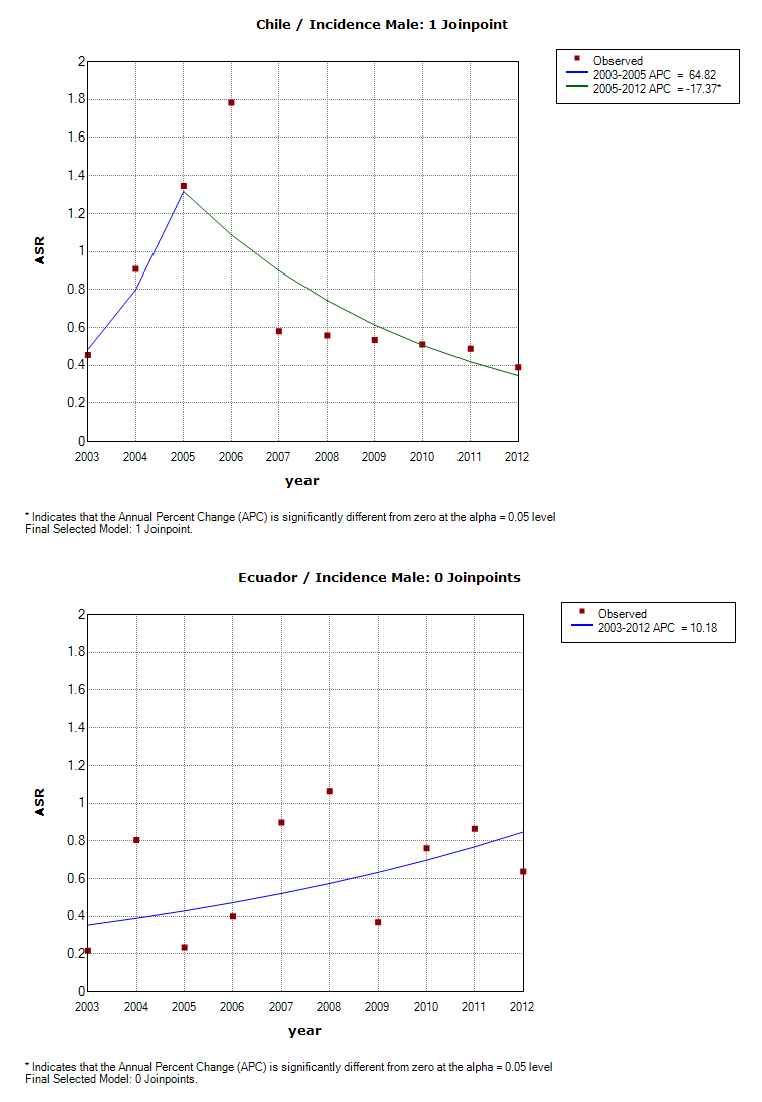

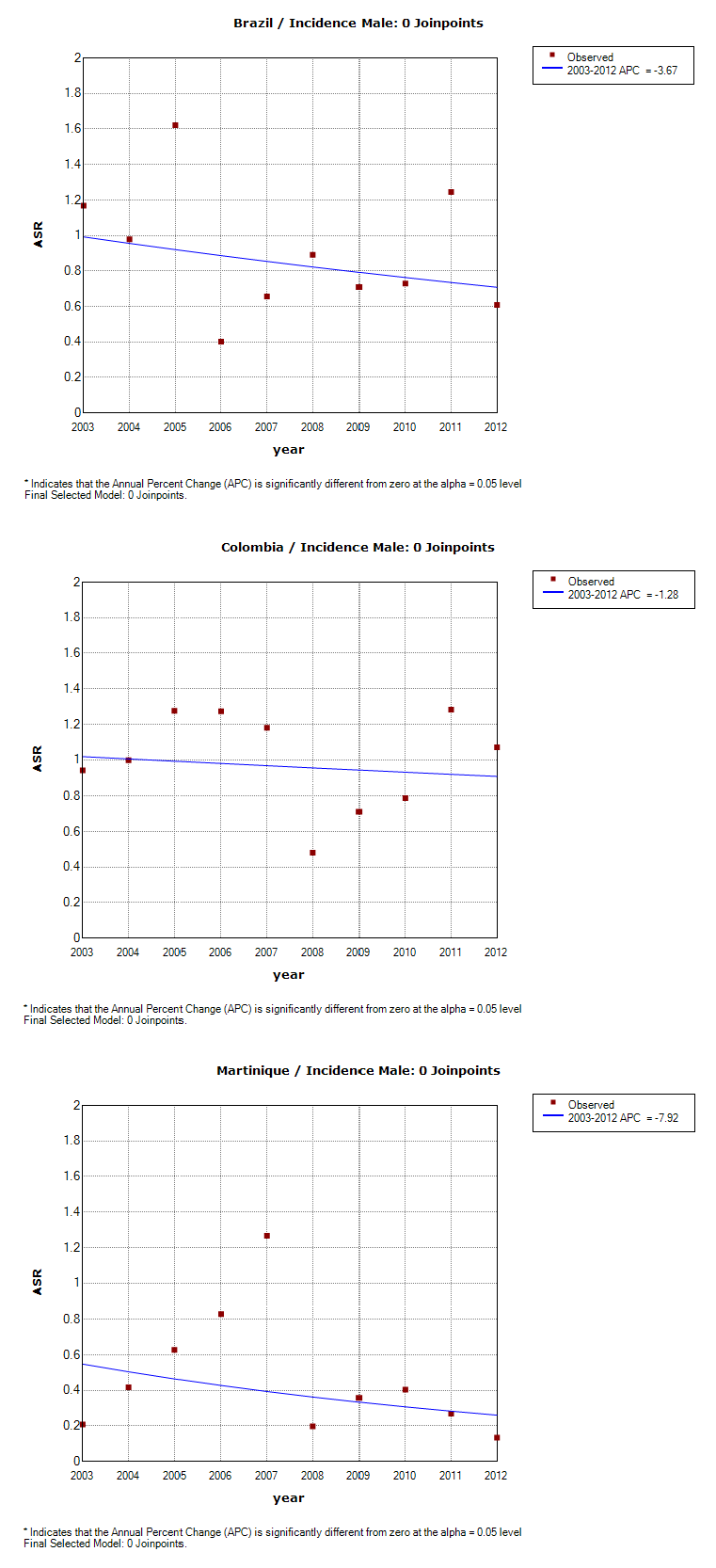


| **Northern Europe** | |
| --- | --- |
| 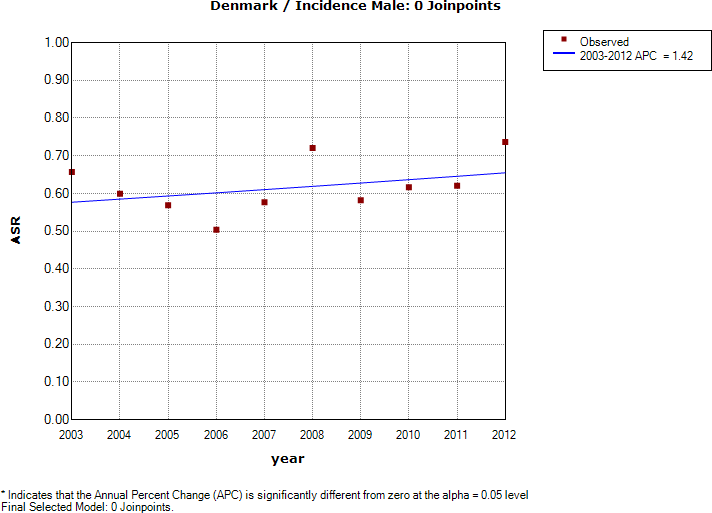 | 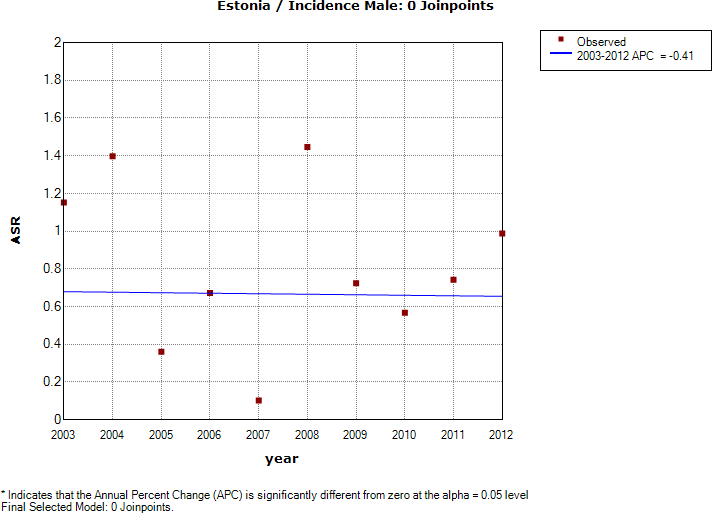 |
|  | 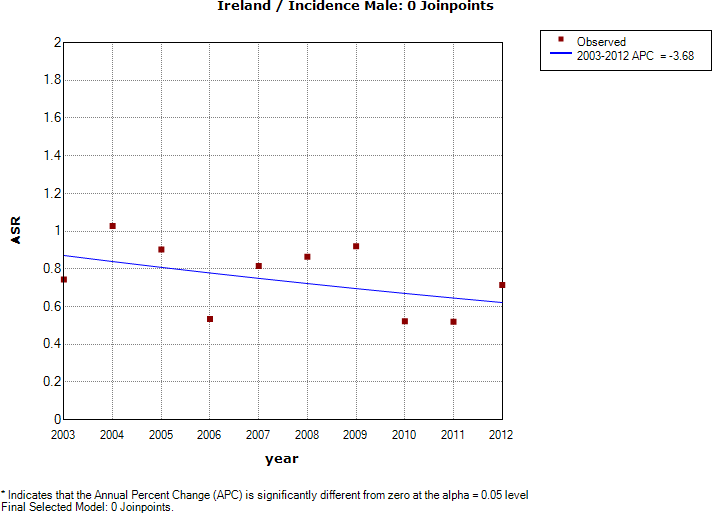 |
|  | 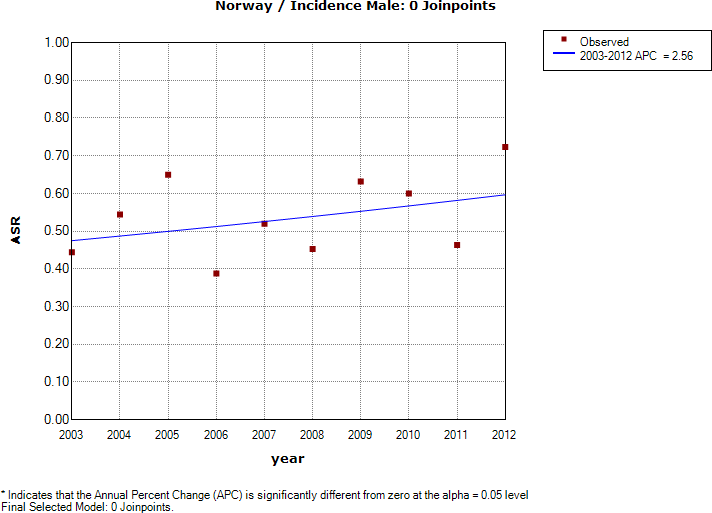 |
|  |  |


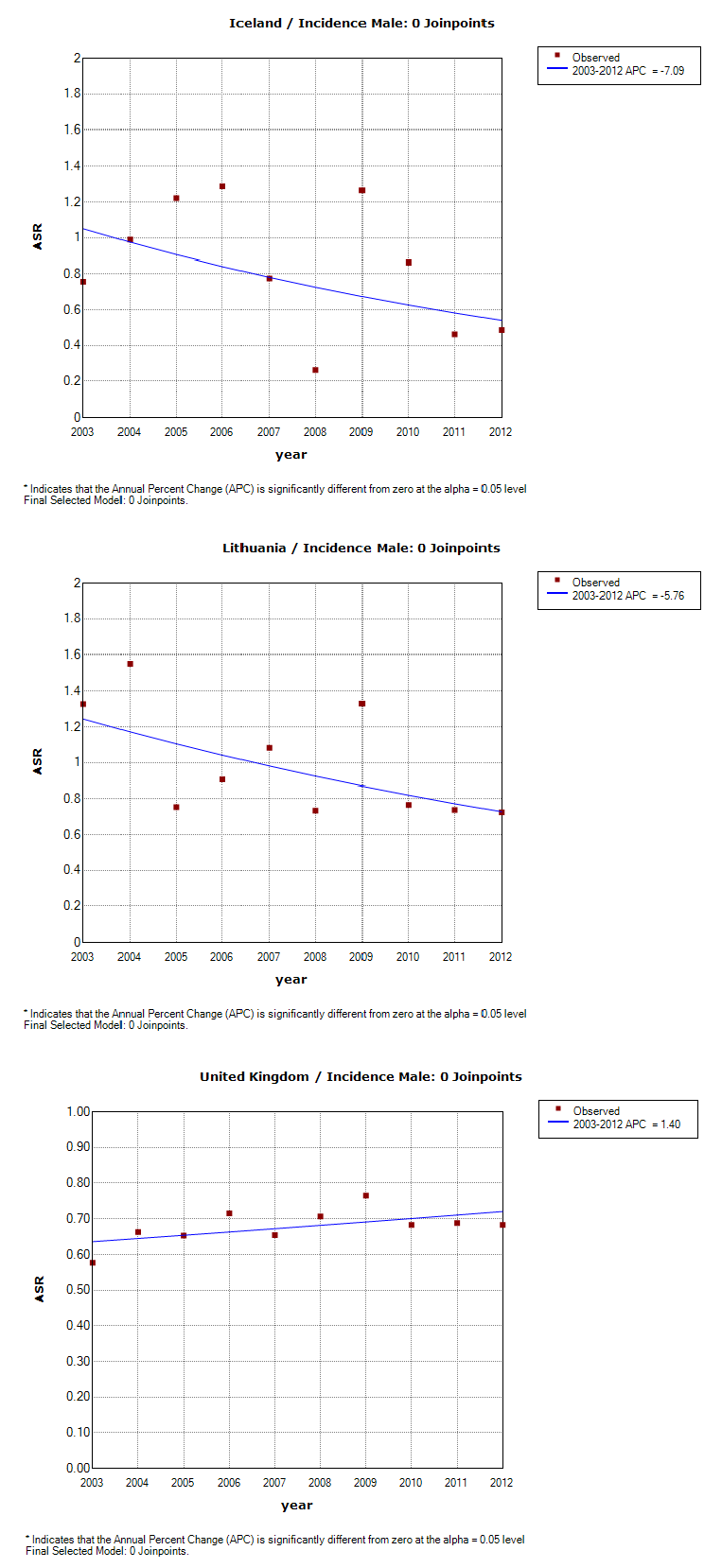


| **Western Europe** | |
| --- | --- |
| 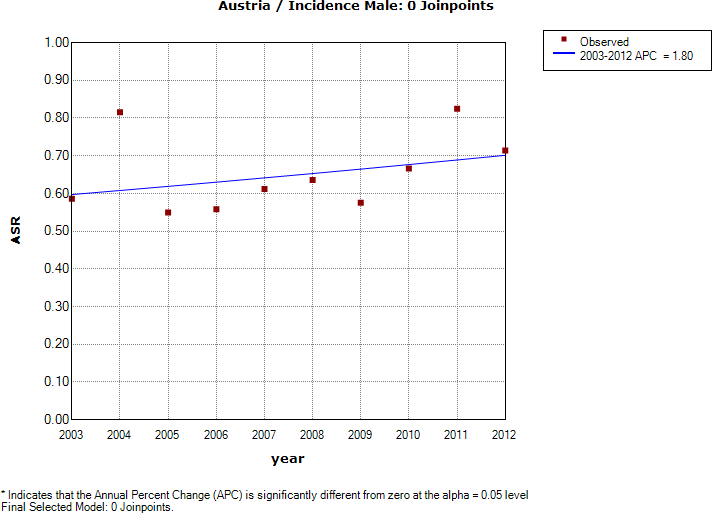 | 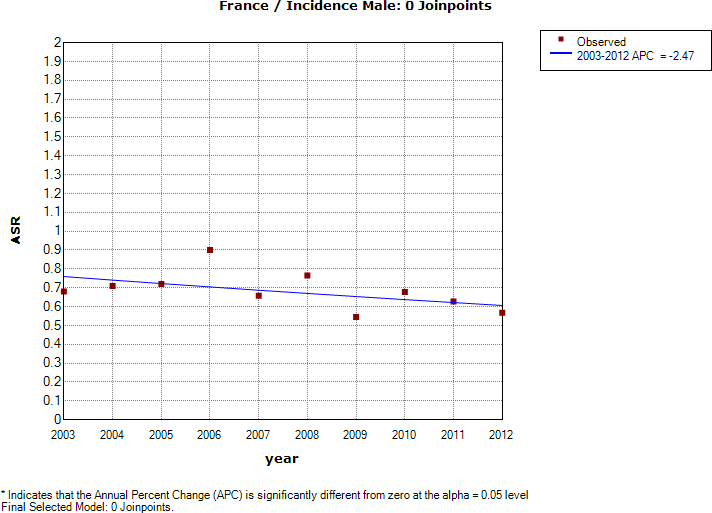 |
| 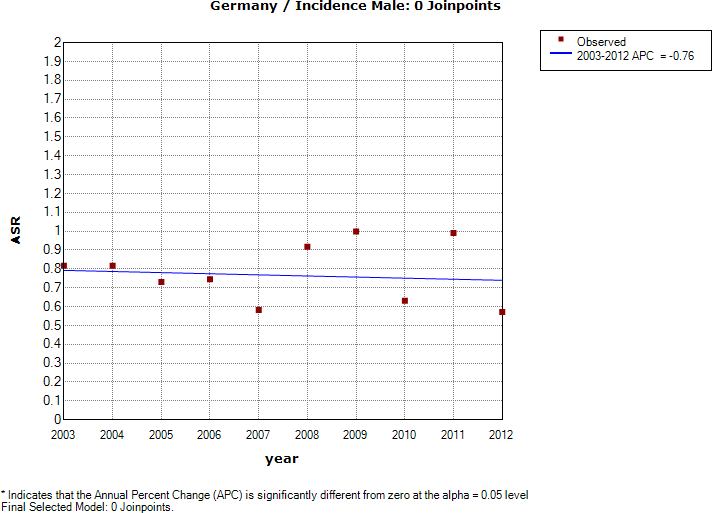 | 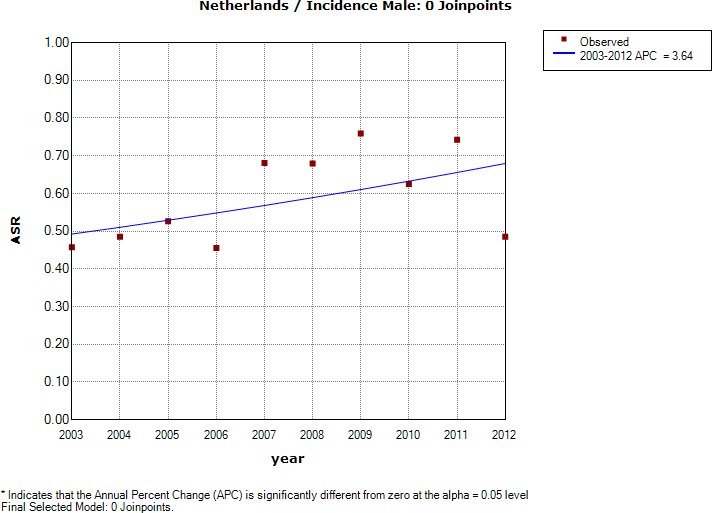 |
| 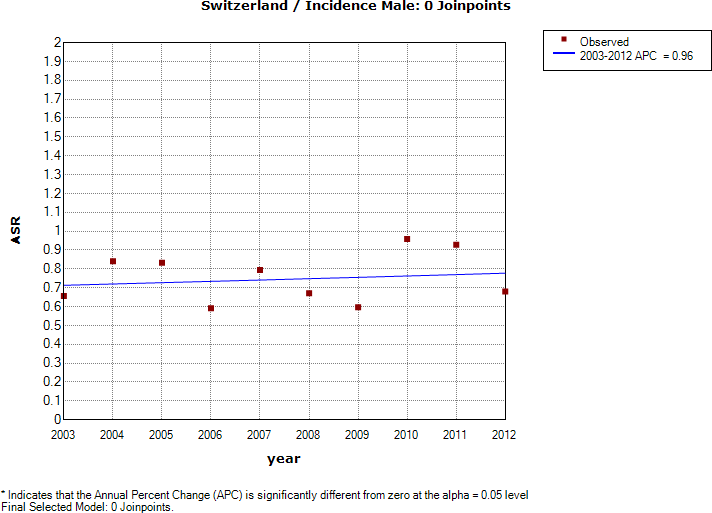 |  |

| **Southern Europe** | |
| --- | --- |
| 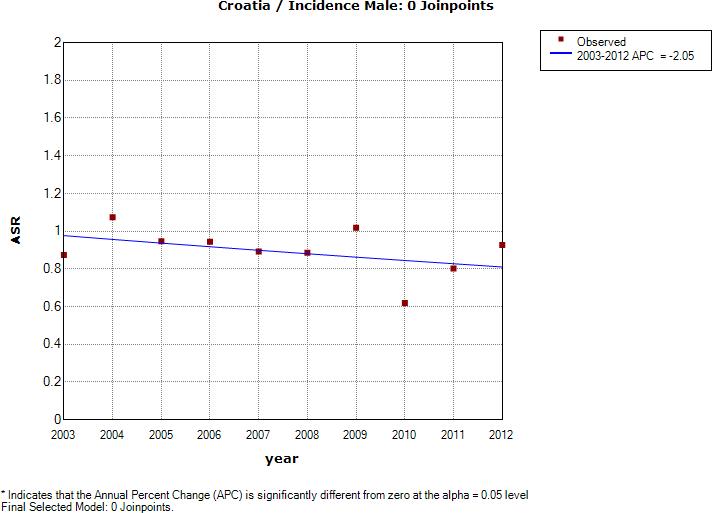 | 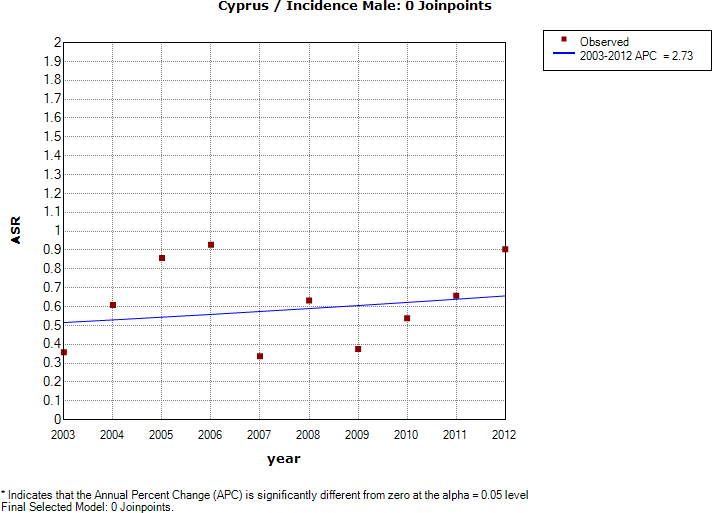 |
|  |  |
|  |  |


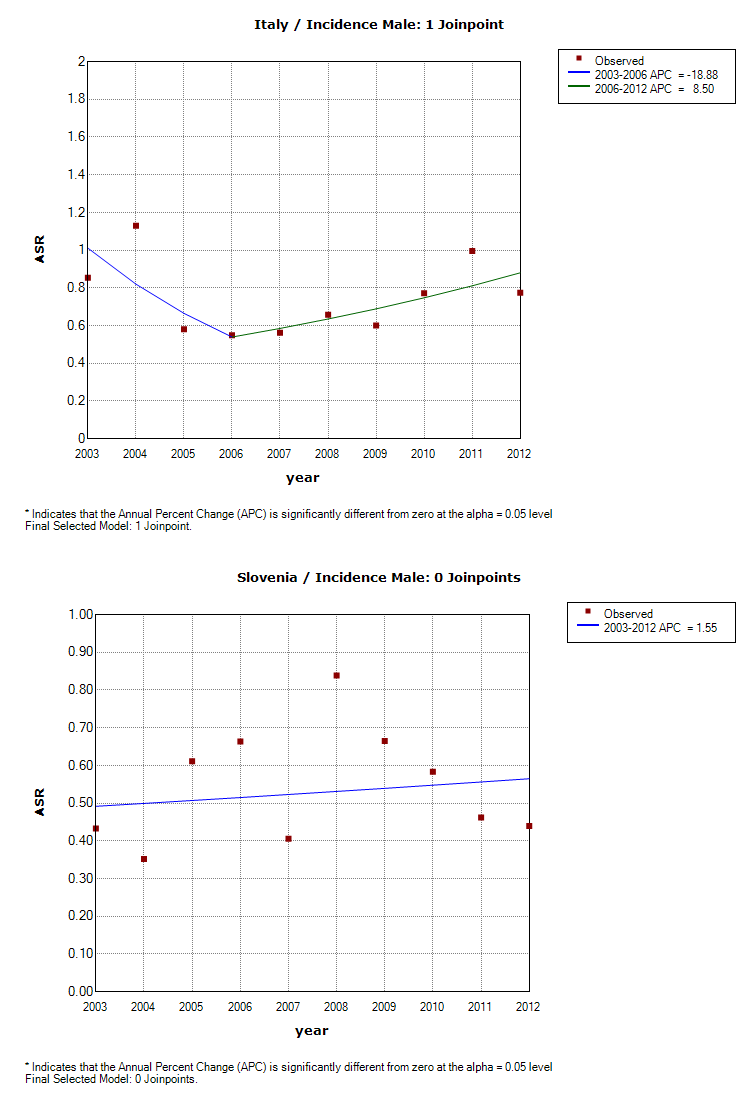

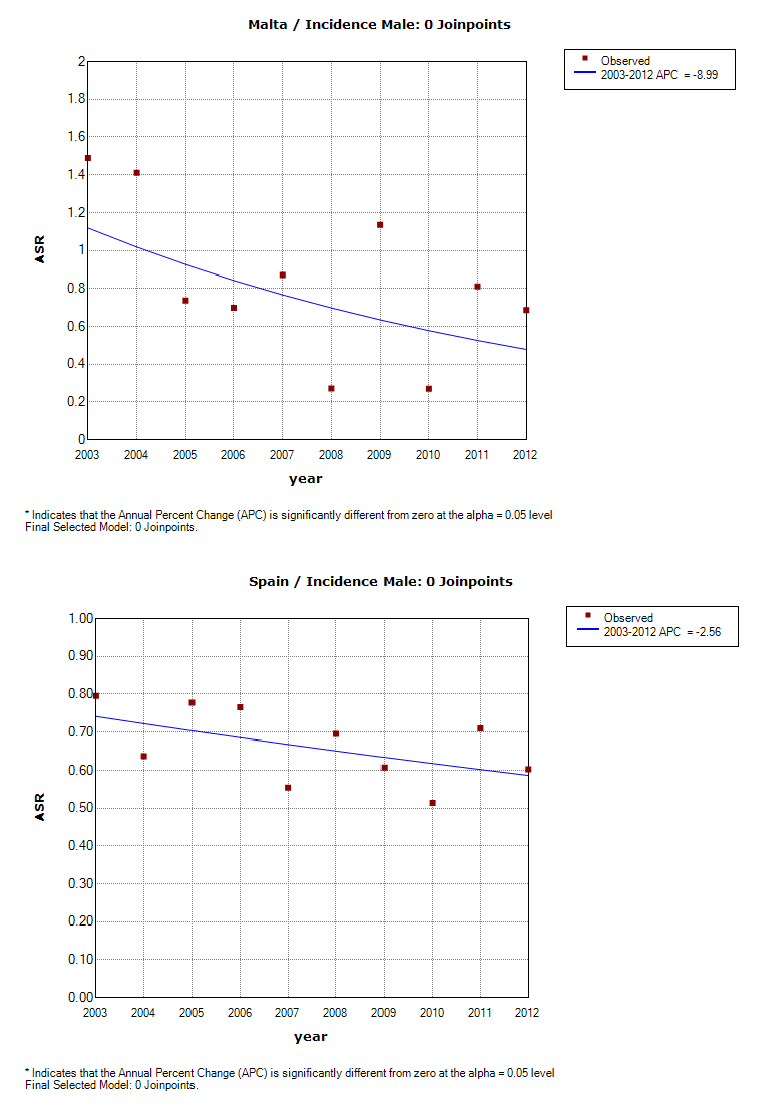


| **Eastern Europe** | |
| --- | --- |
| 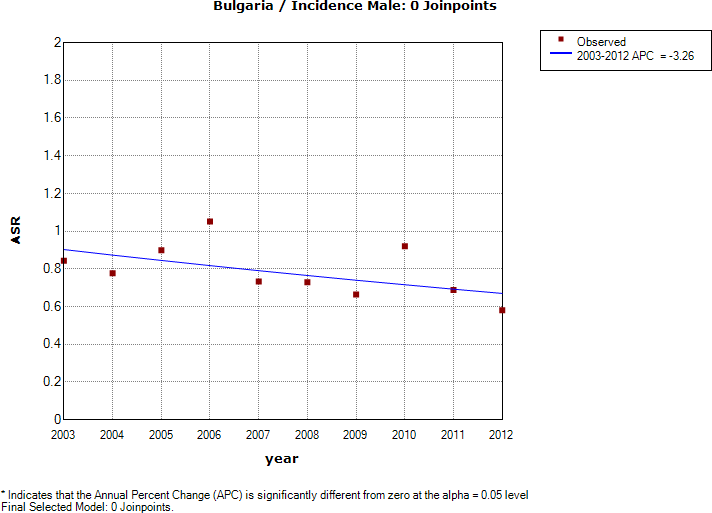 | 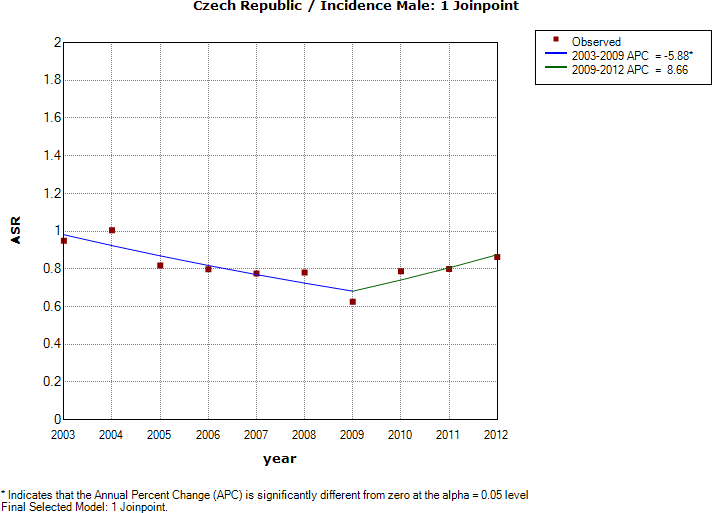 |
| 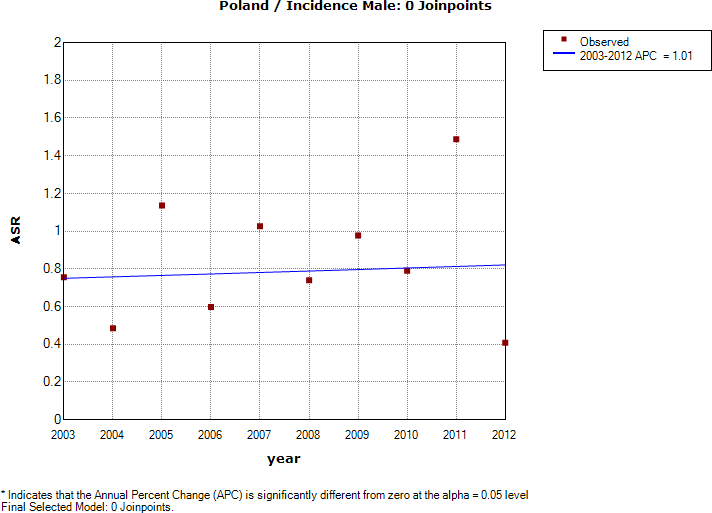 |  |
| **Africa** | |
| 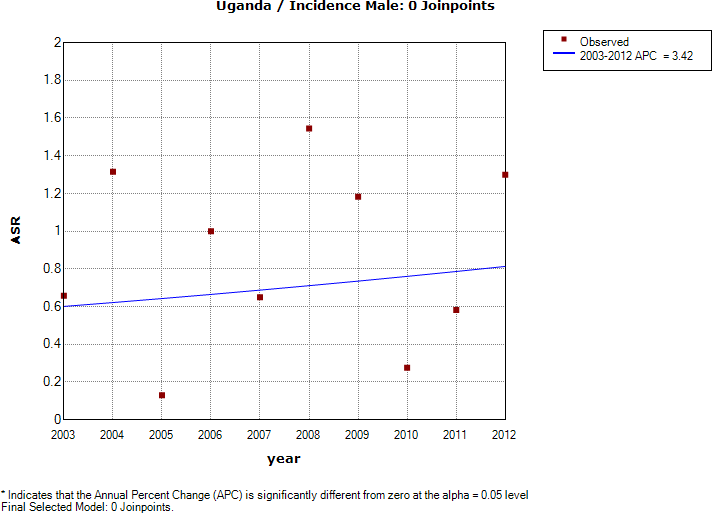 |  |


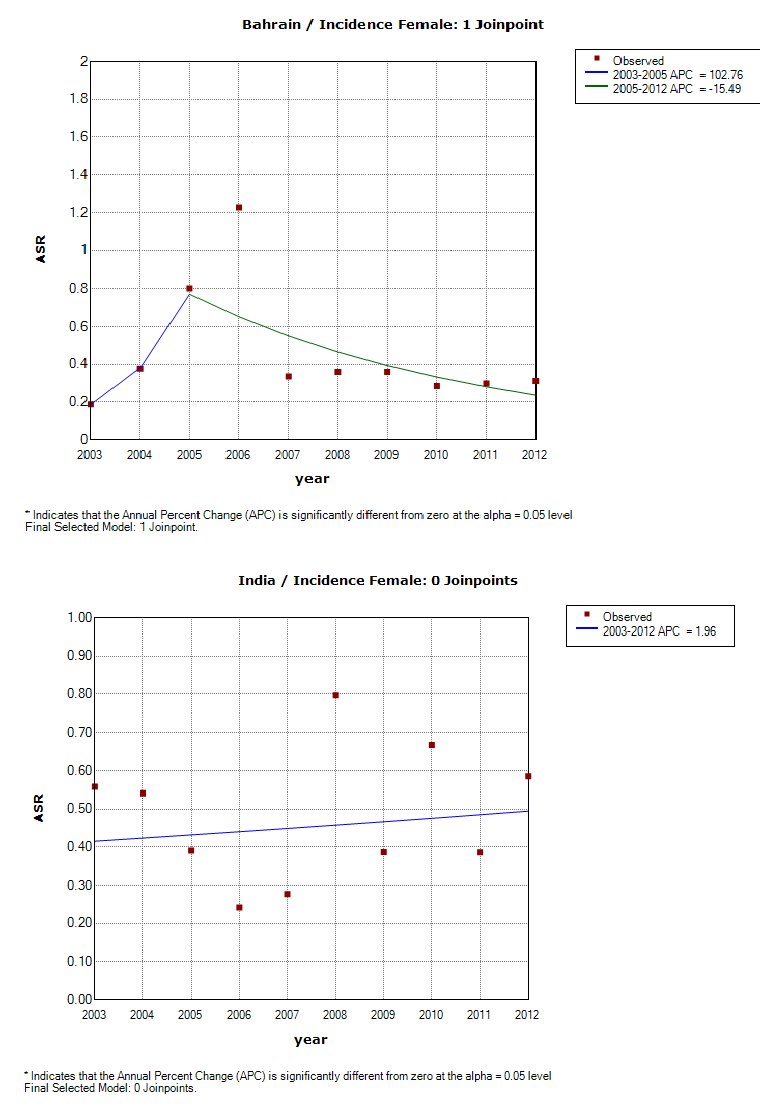

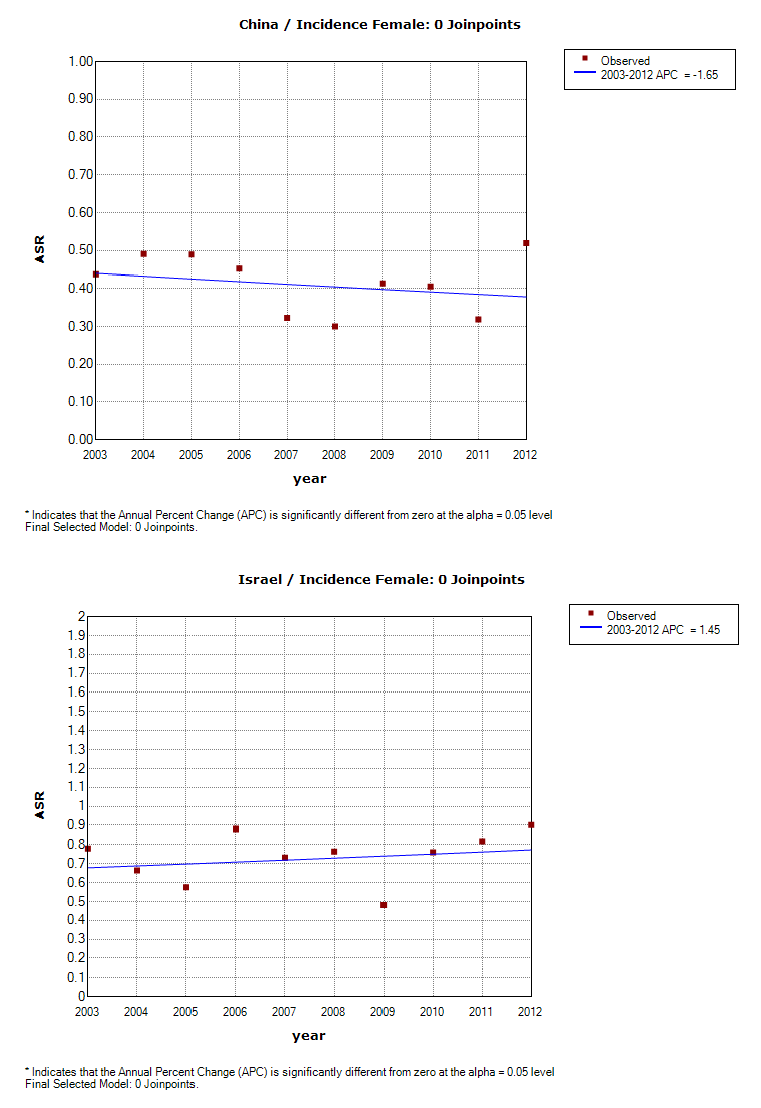
b.) Female

| **Asia** | |
| --- | --- |
|  |  |
|  |  |
| 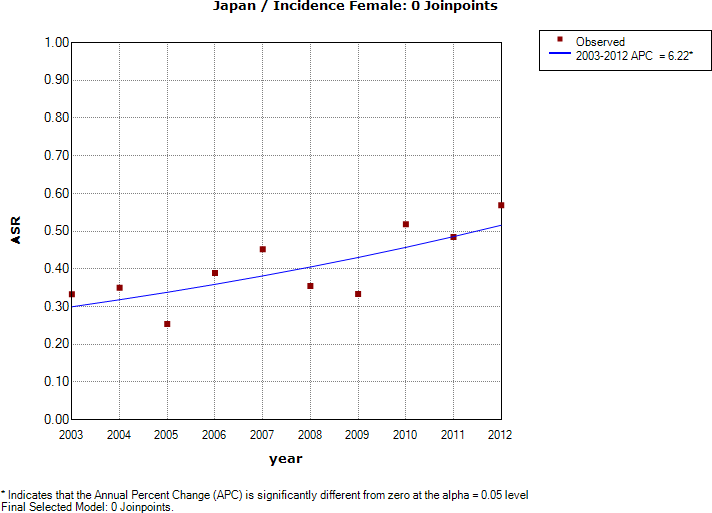 | 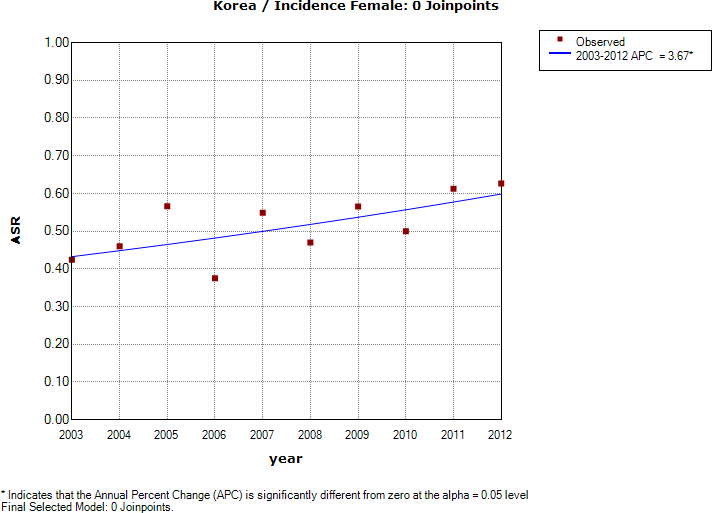 |

|  | 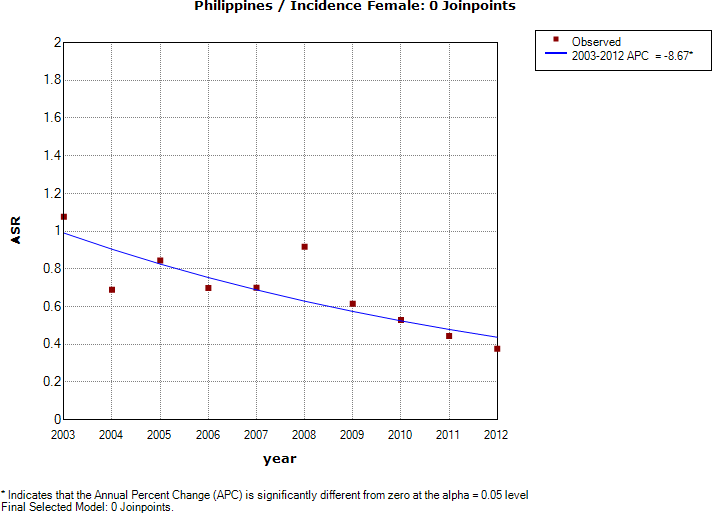 |
| --- | --- |
|  | 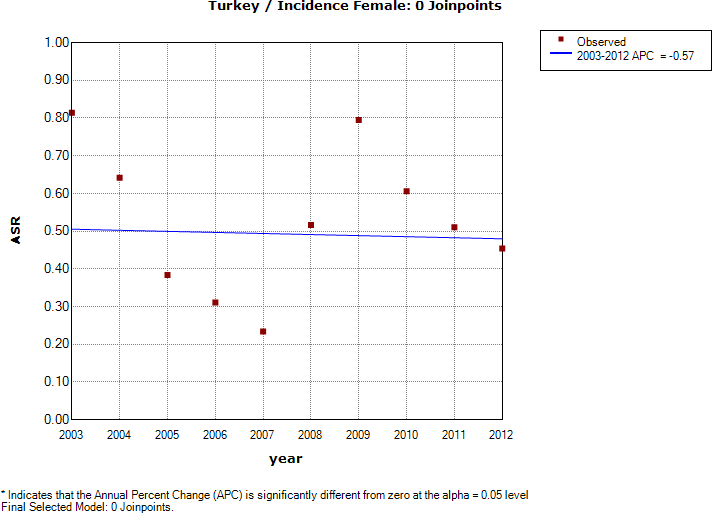 |
| **Oceania** | |
| 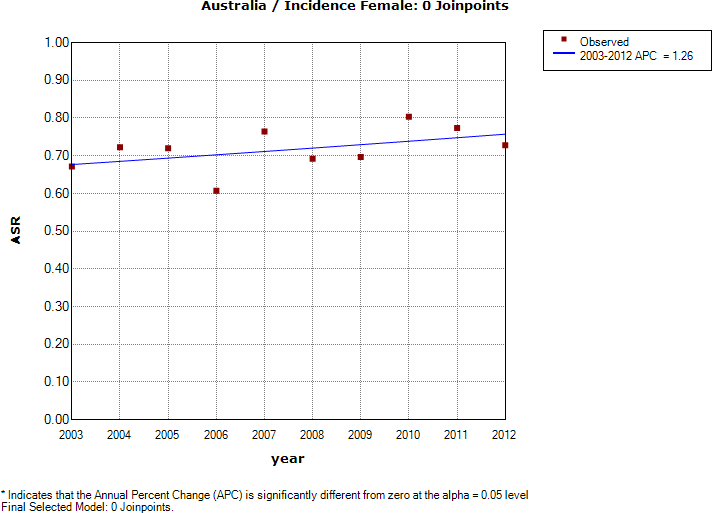 | 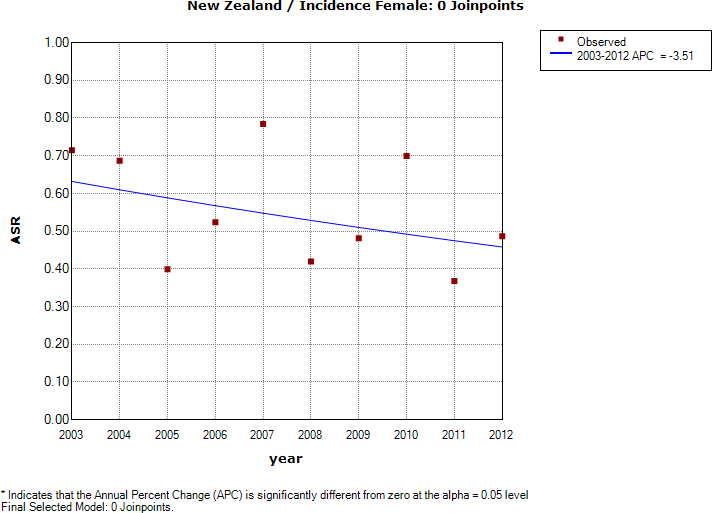 |


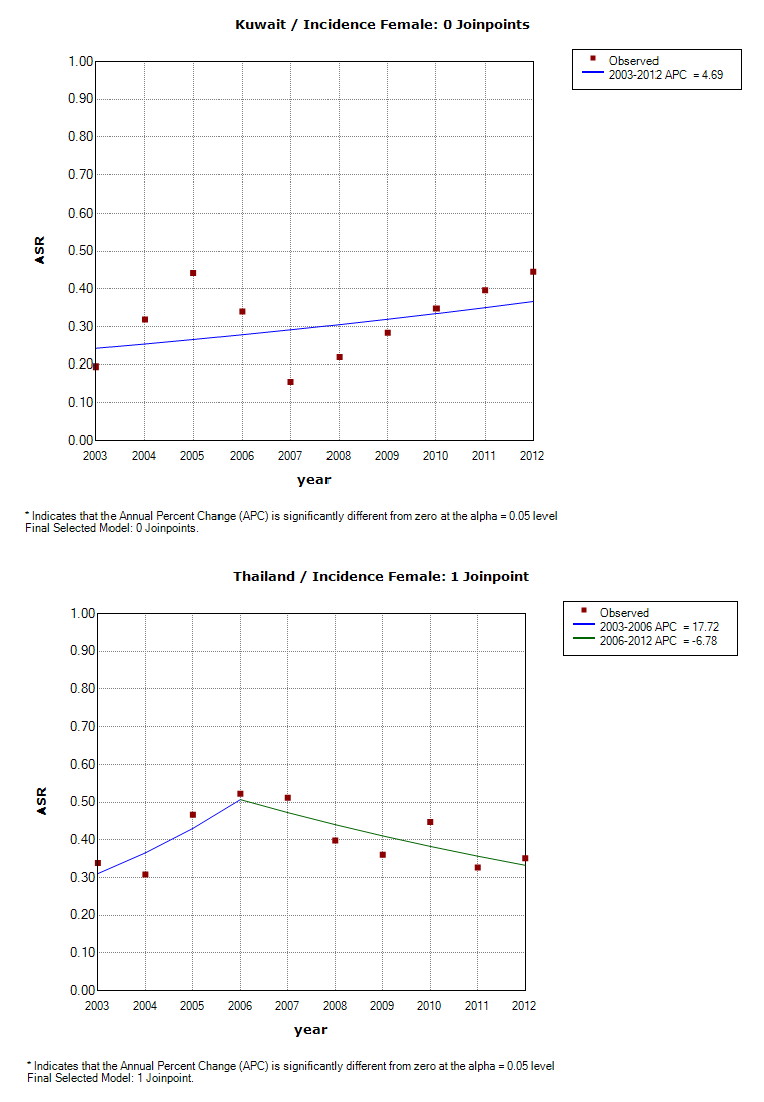


| **Northern America** | |
| --- | --- |
| 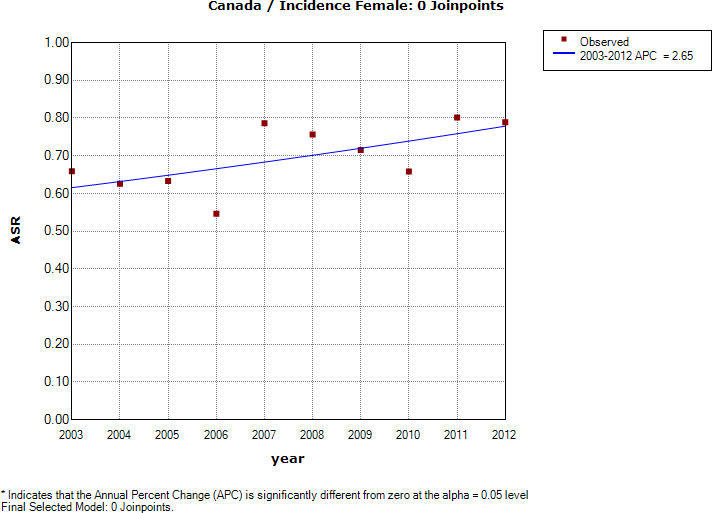 | 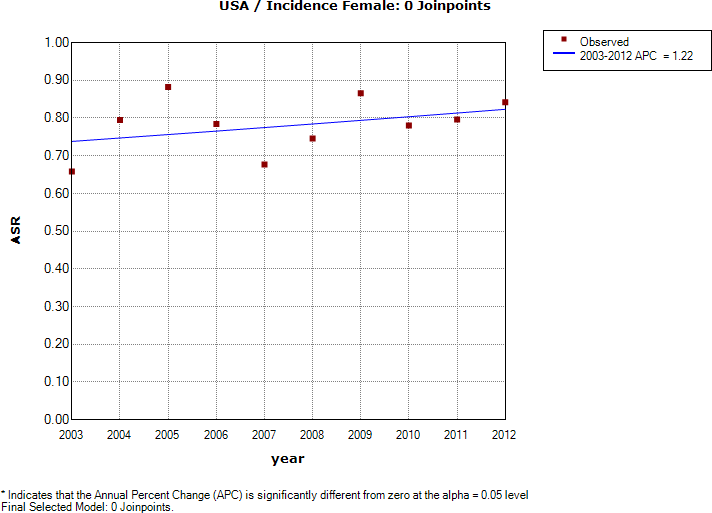 |
| **Southern America** | |
| 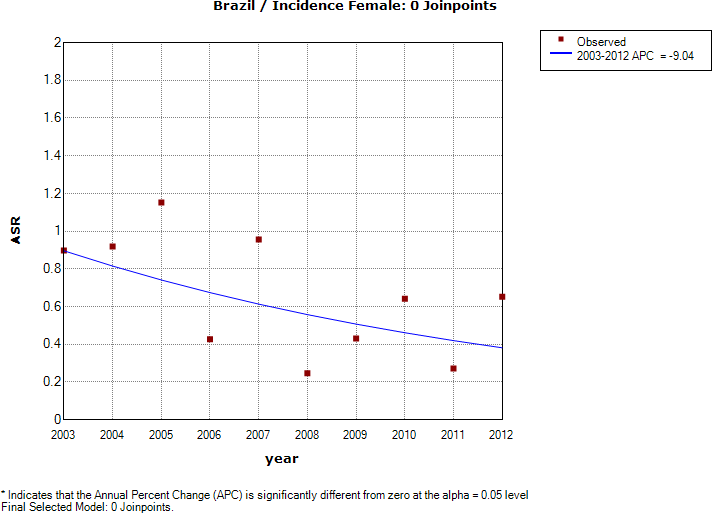 | 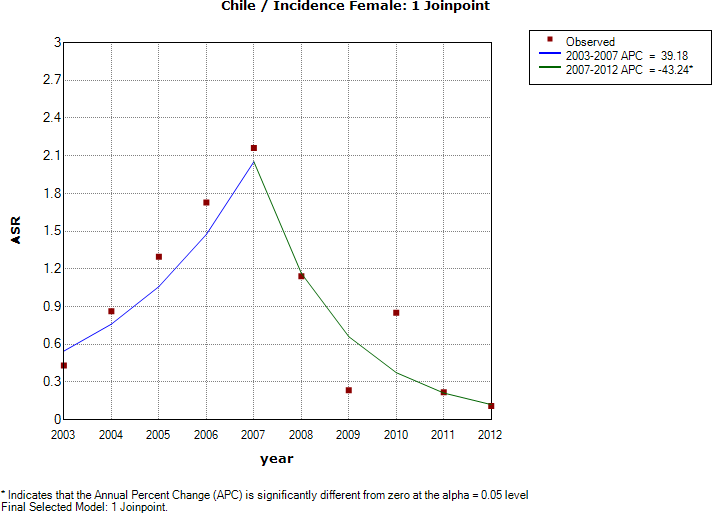 |
| 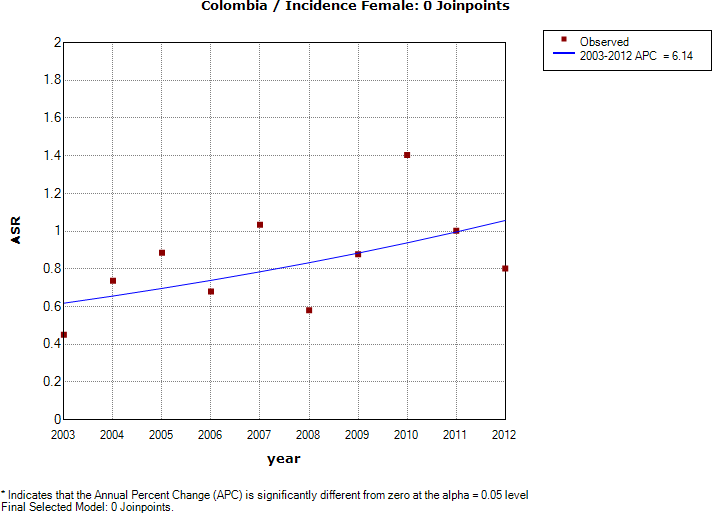 | 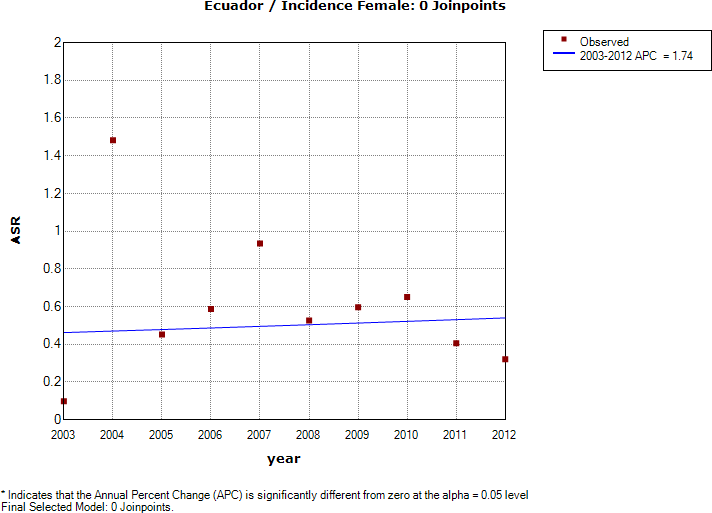 |
| 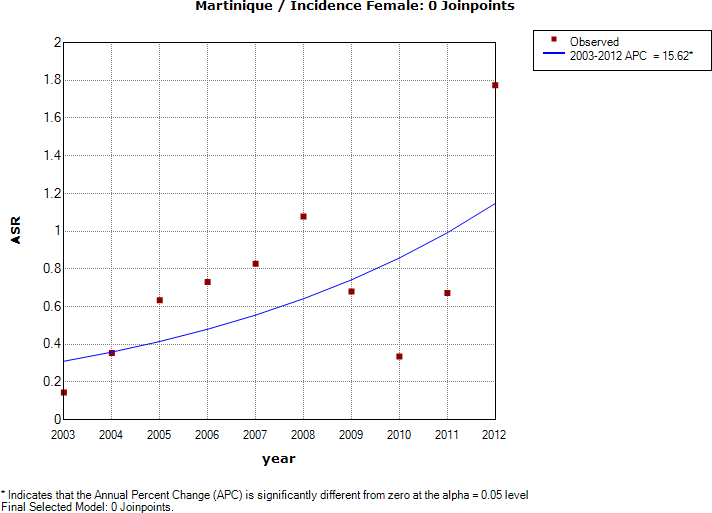 |  |

| **Northern Europe** | |
| --- | --- |
| 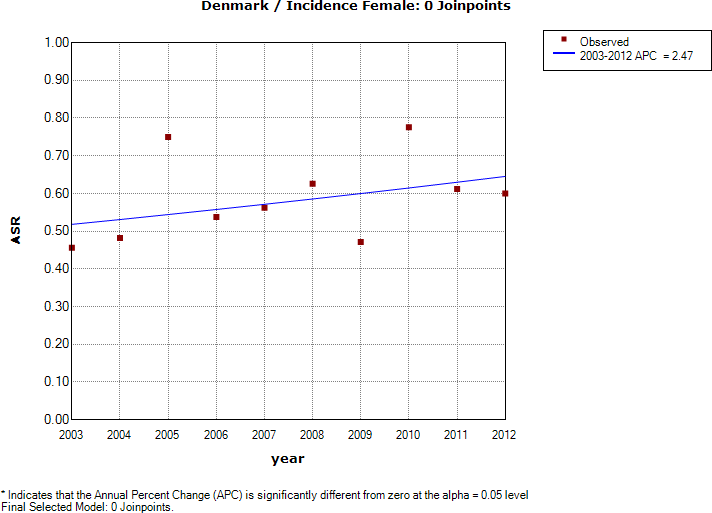 | 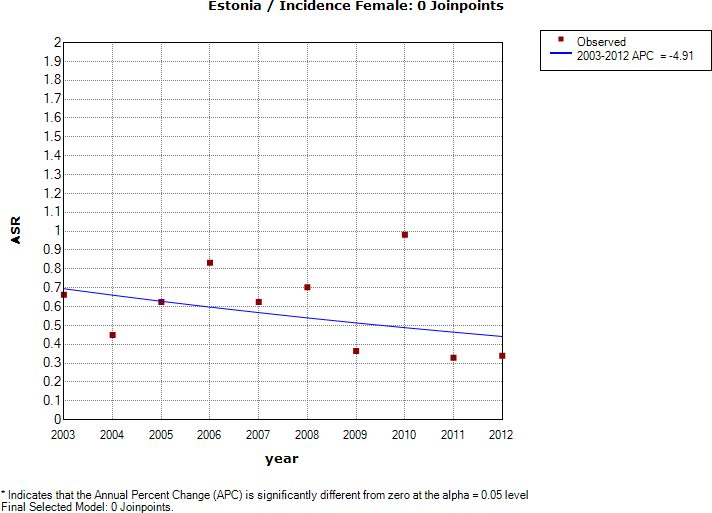 |
| 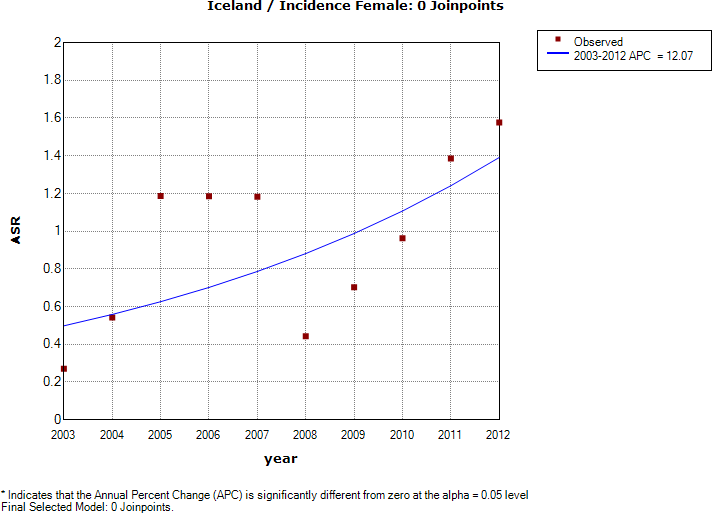 | 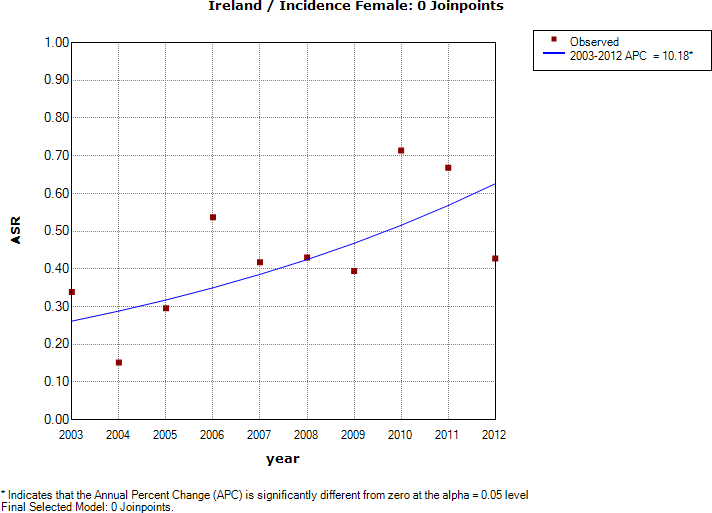 |
| 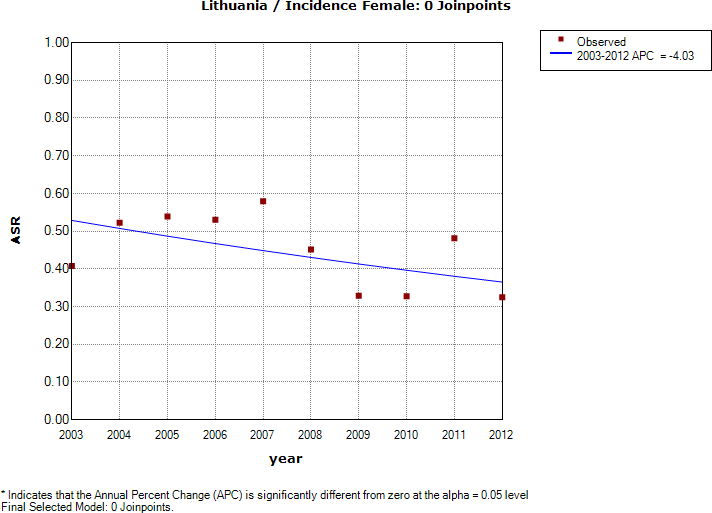 | 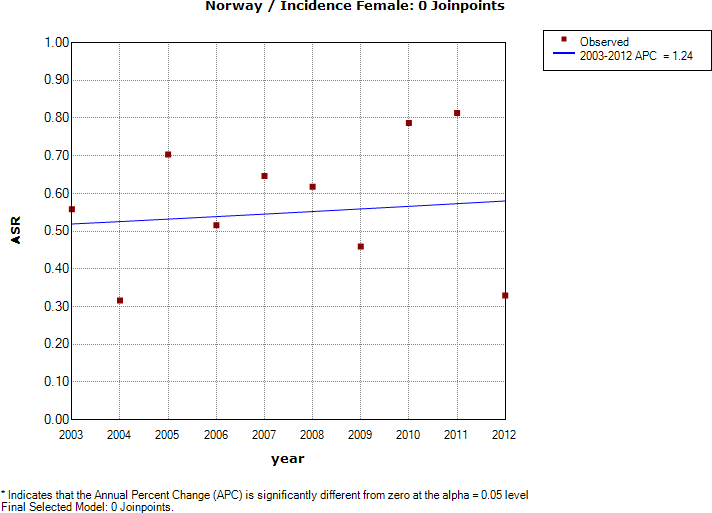 |

| 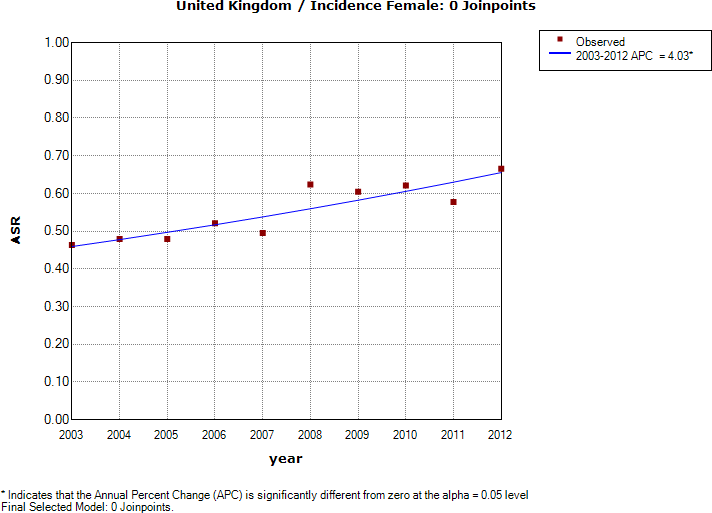 |  |
| --- | --- |
| **Western Europe** | |
| 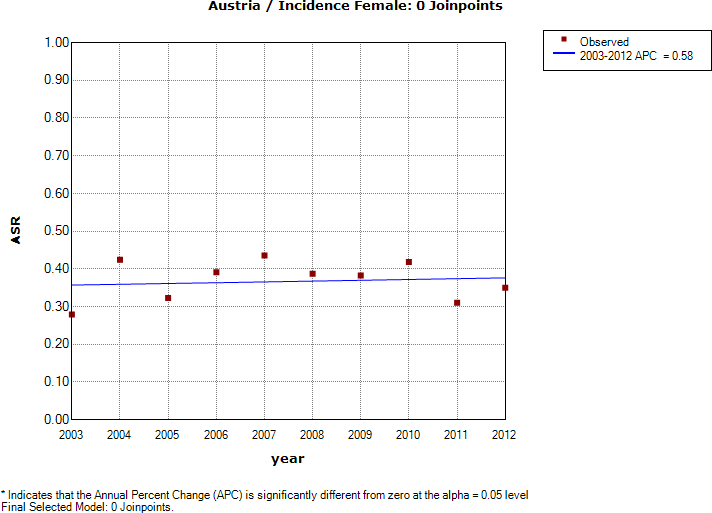 | 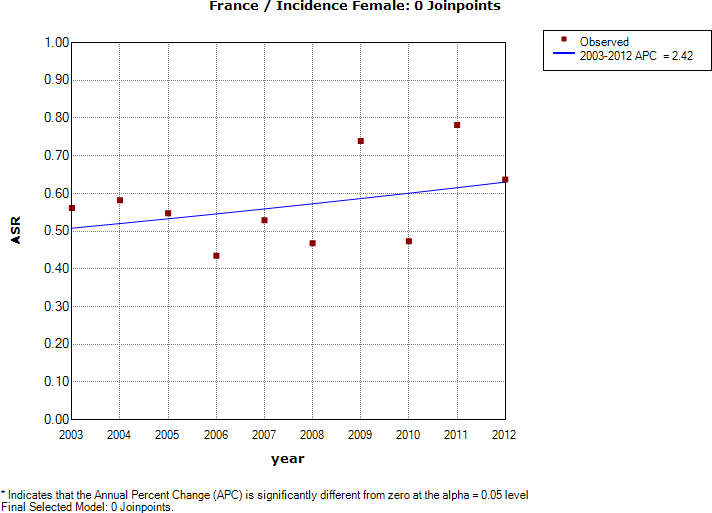 |
| 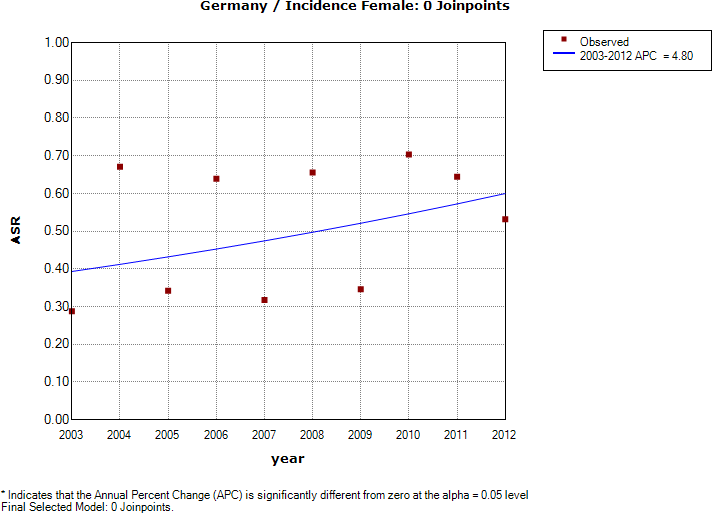 | 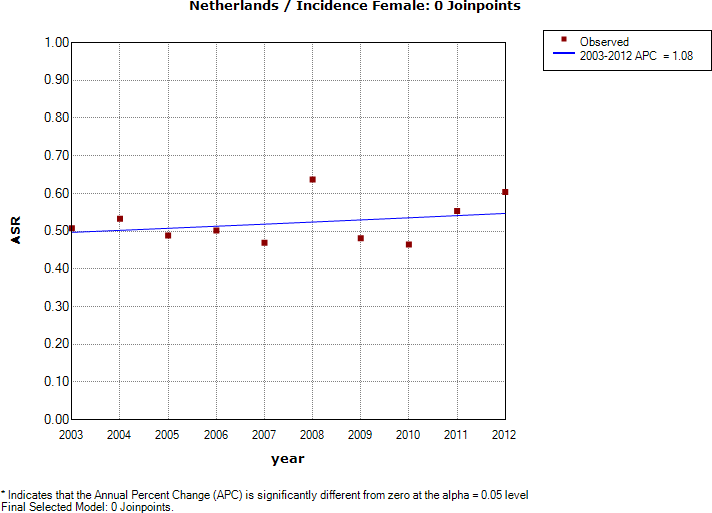 |

| 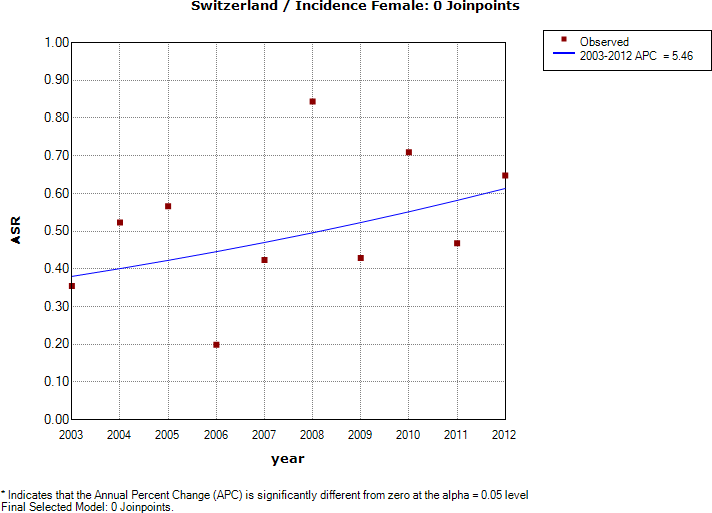 |  |
| --- | --- |
| **Southern Europe** | |
| 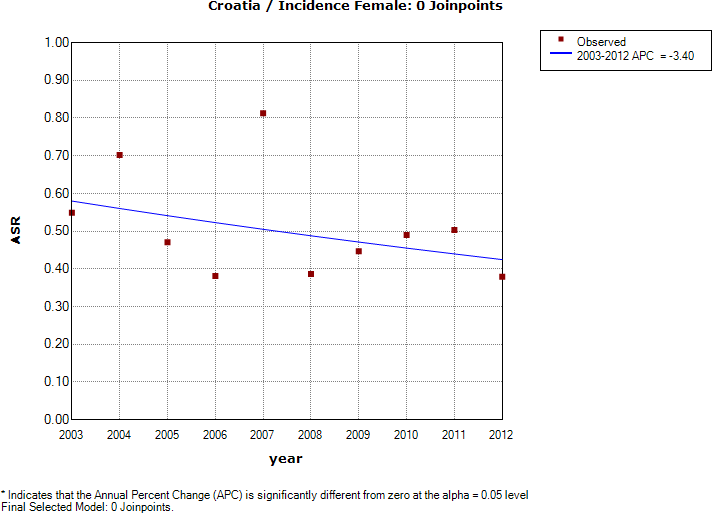 | 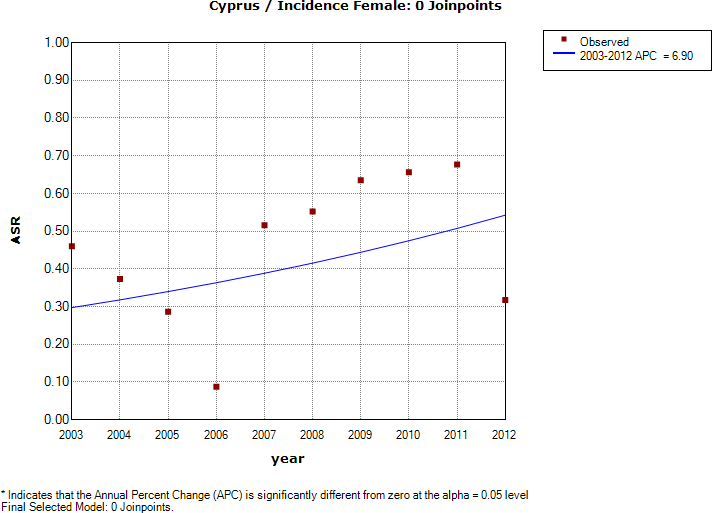 |
| 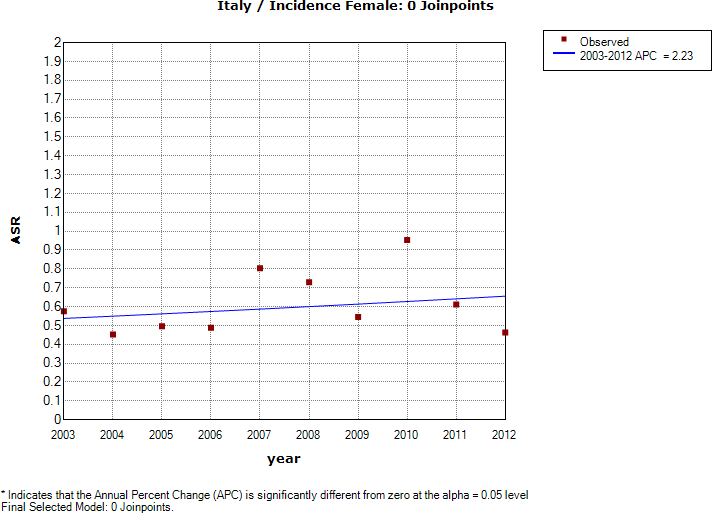 | 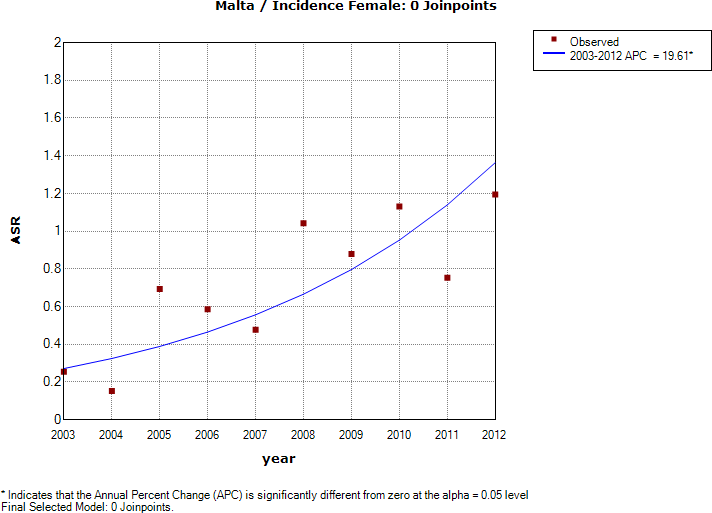 |
| 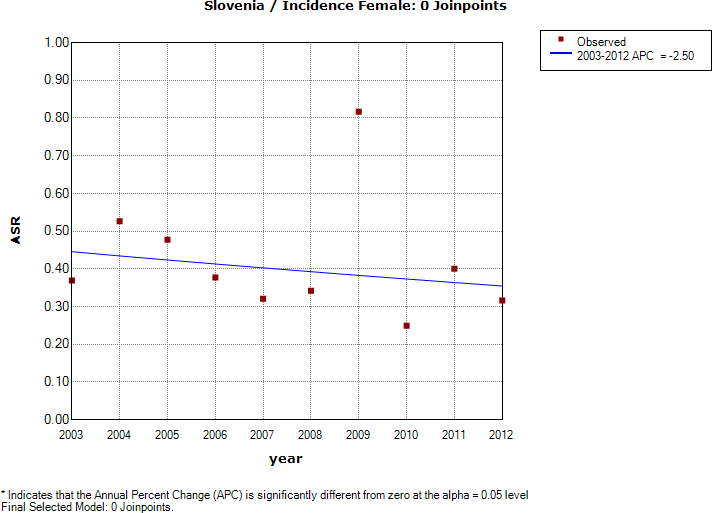 | 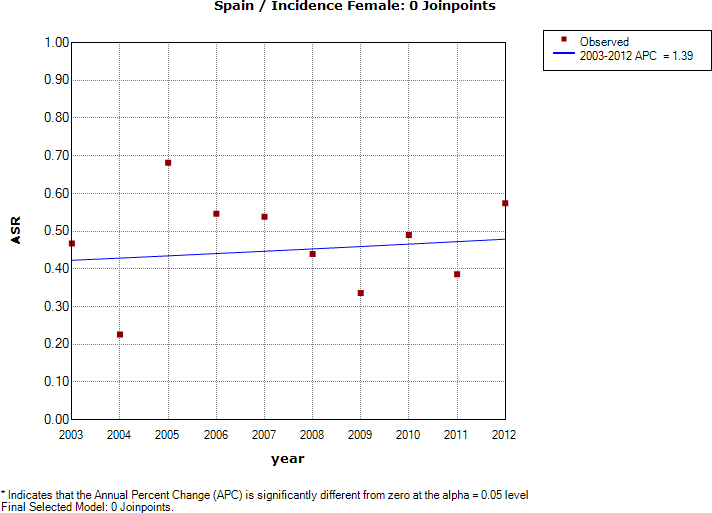 |

| **Eastern Europe** | |
| --- | --- |
| 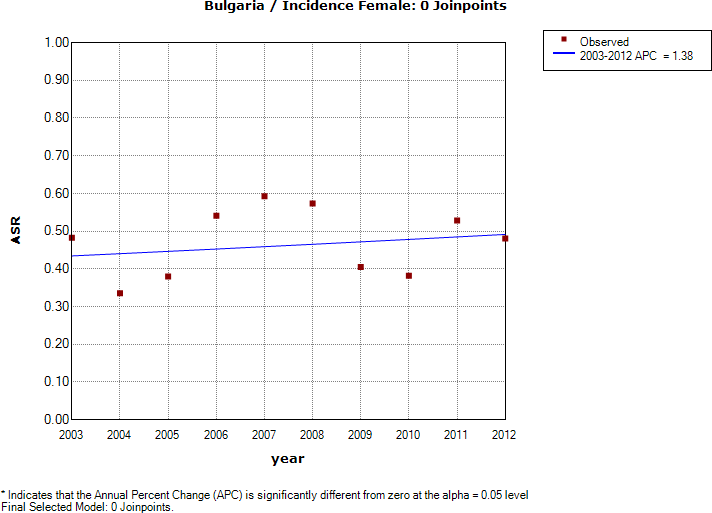 | 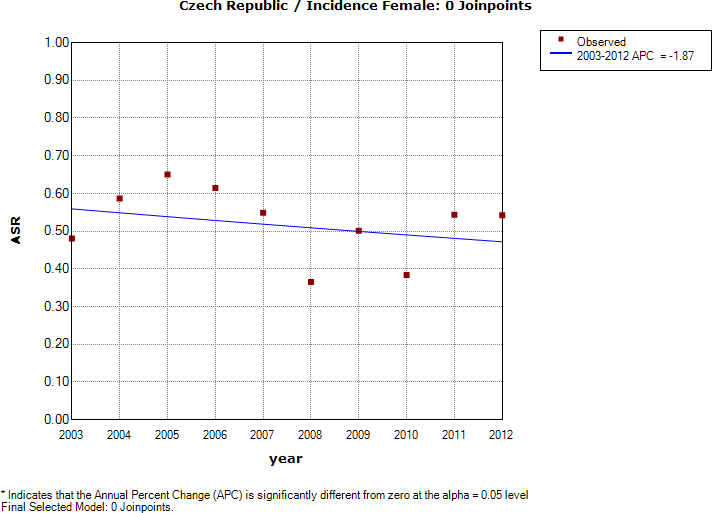 |
| 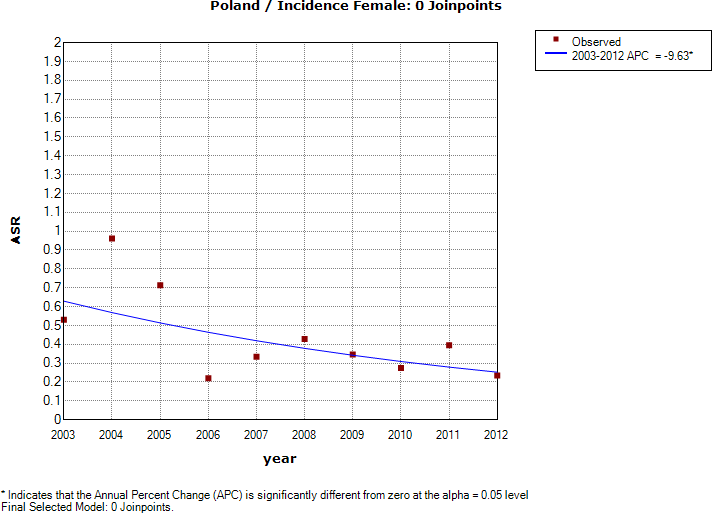 |  |
| **Africa** | |
| 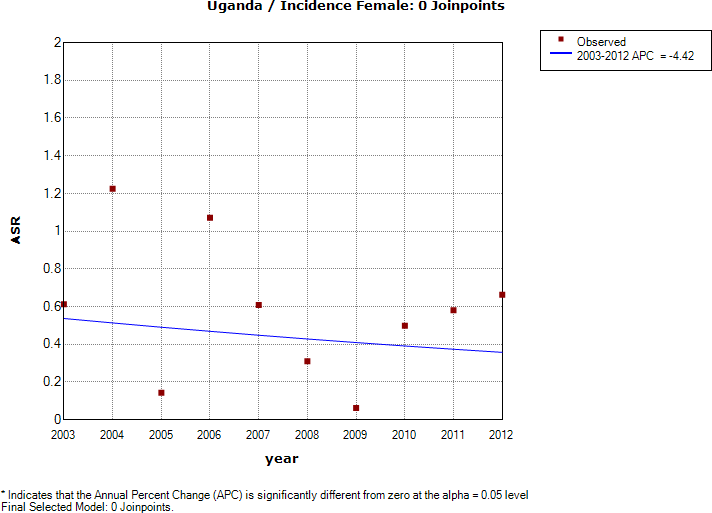 |  |

###
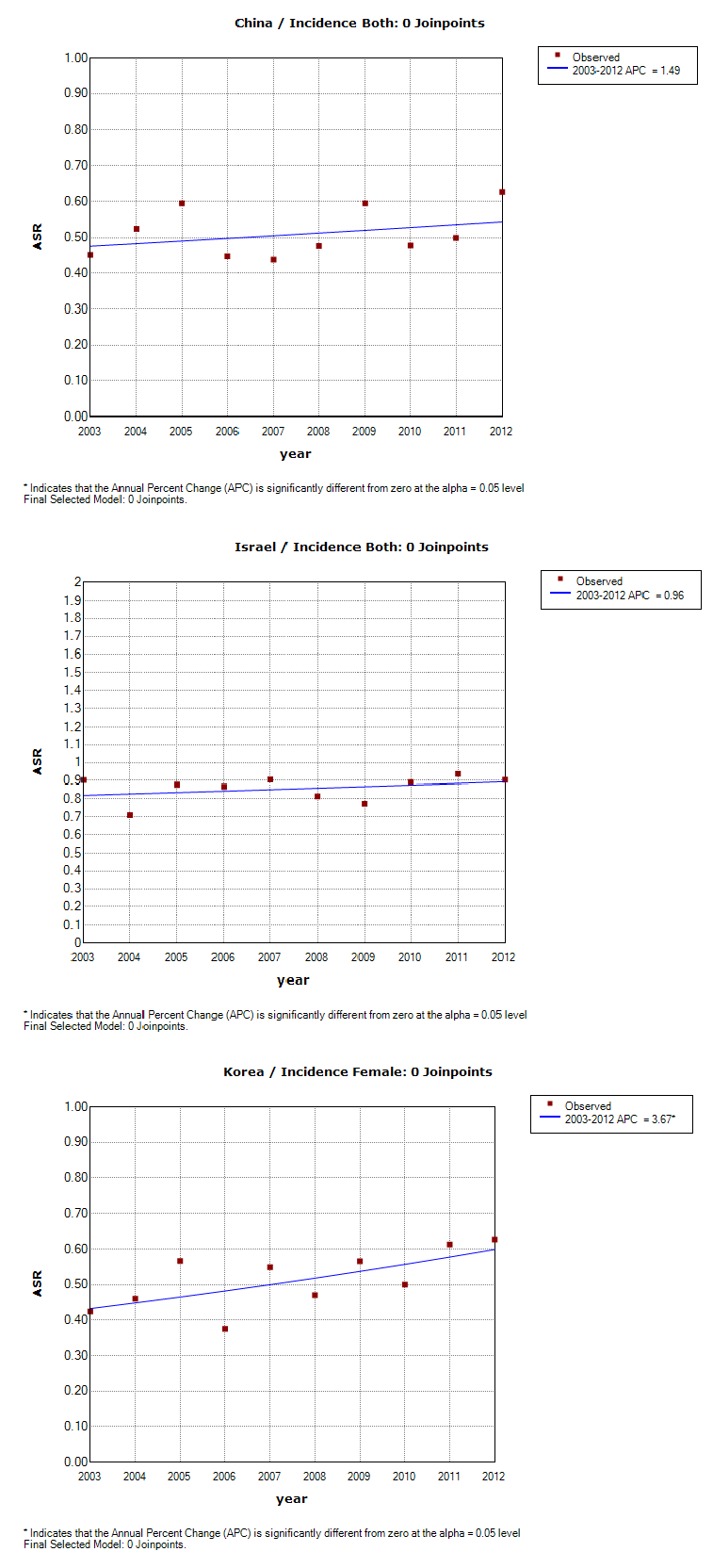

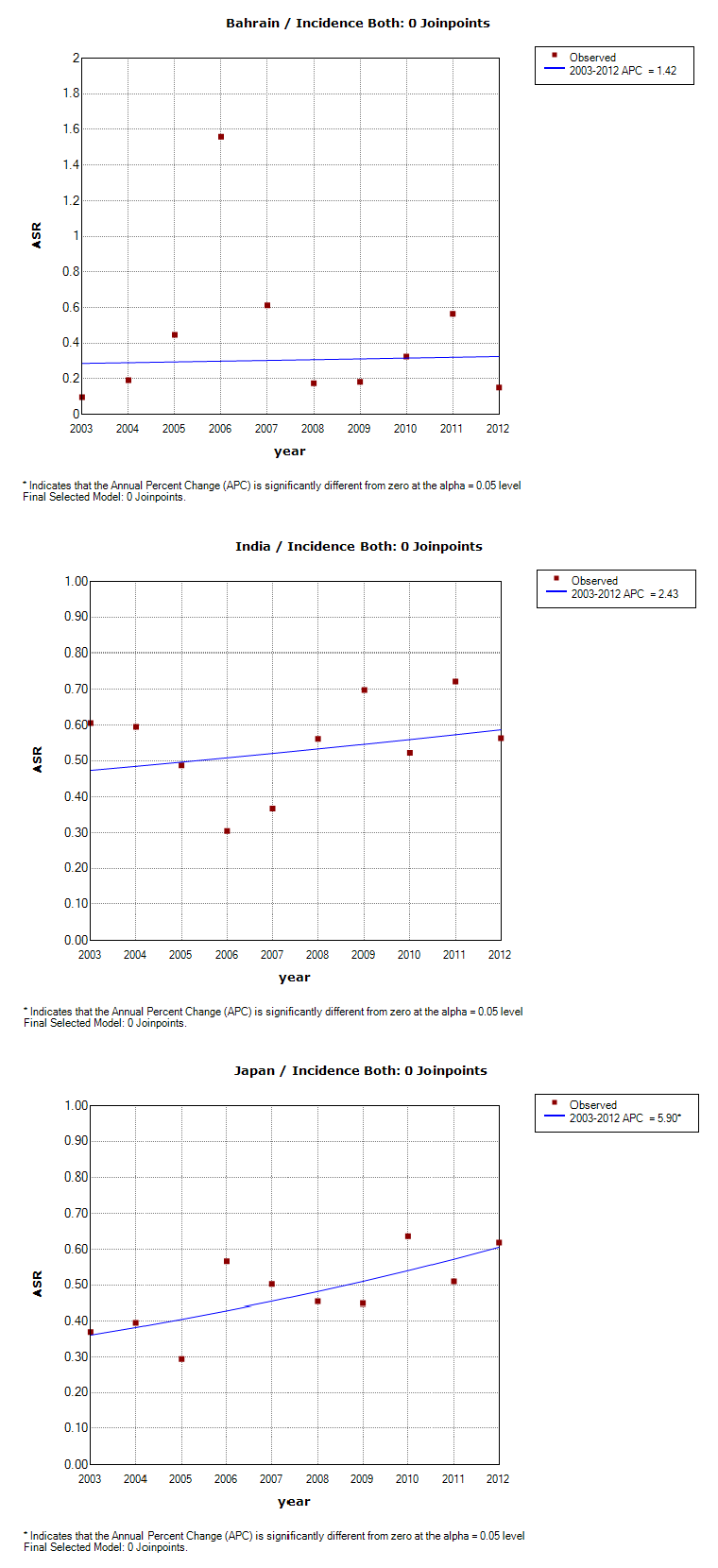
c.) Both

| **Asia** | |
| --- | --- |
|  |  |
|  |  |
|  |  |

|  | 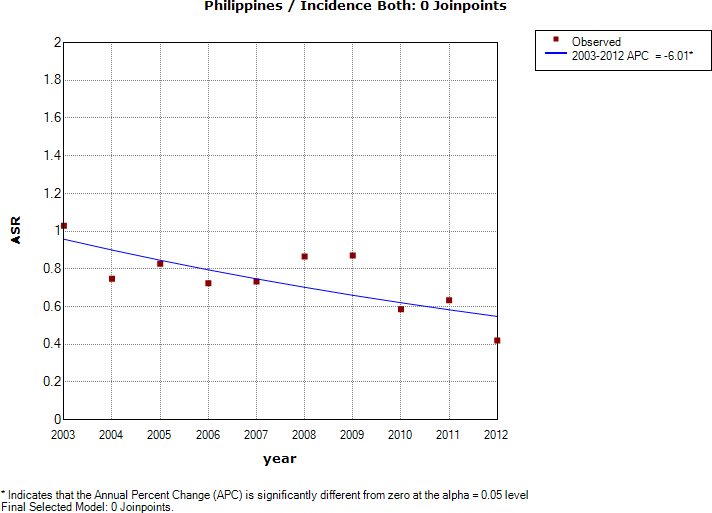 |
| --- | --- |
|  | 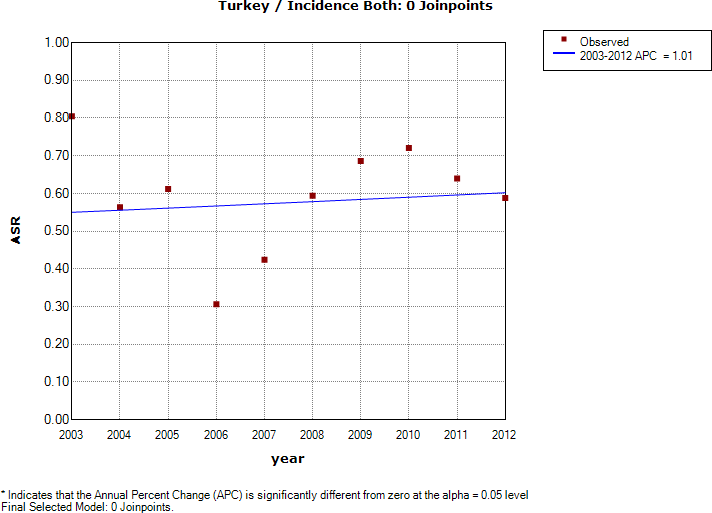 |
| **Oceania** | |
| 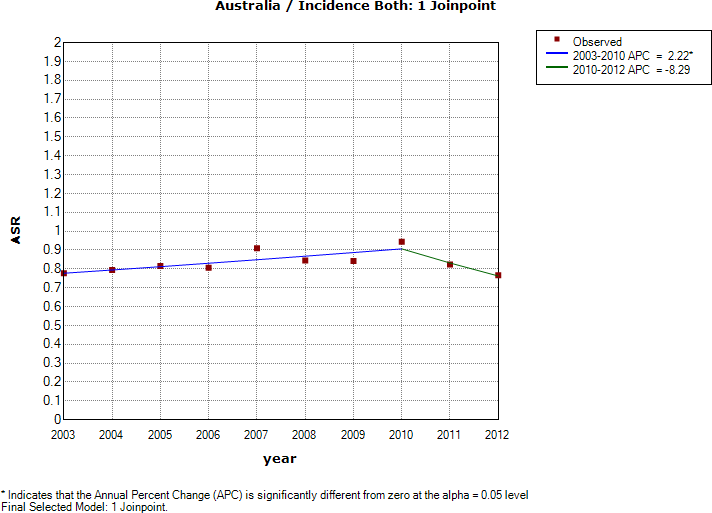 | 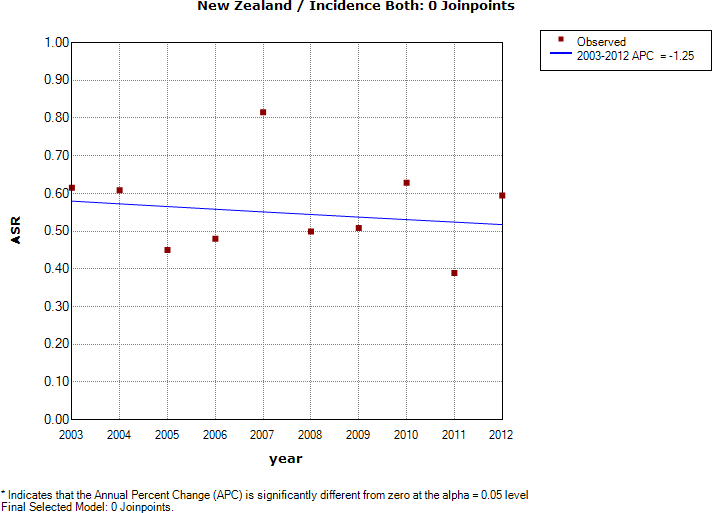 |


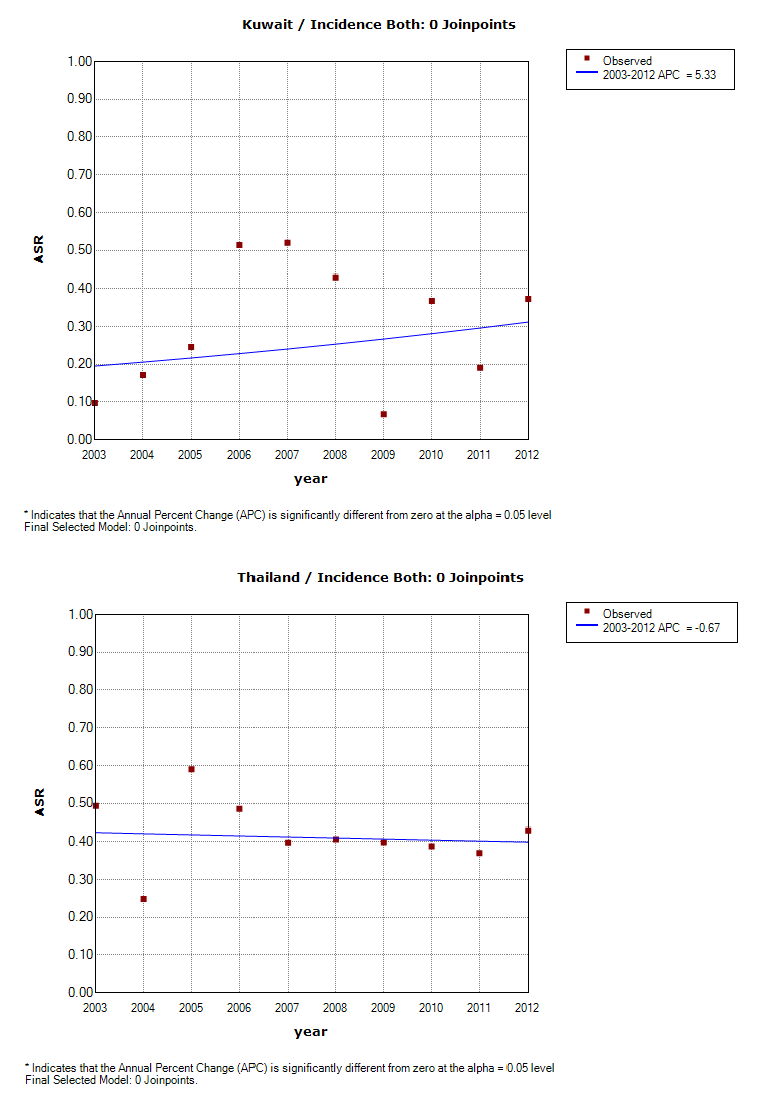


| **Northern America** | |
| --- | --- |
| 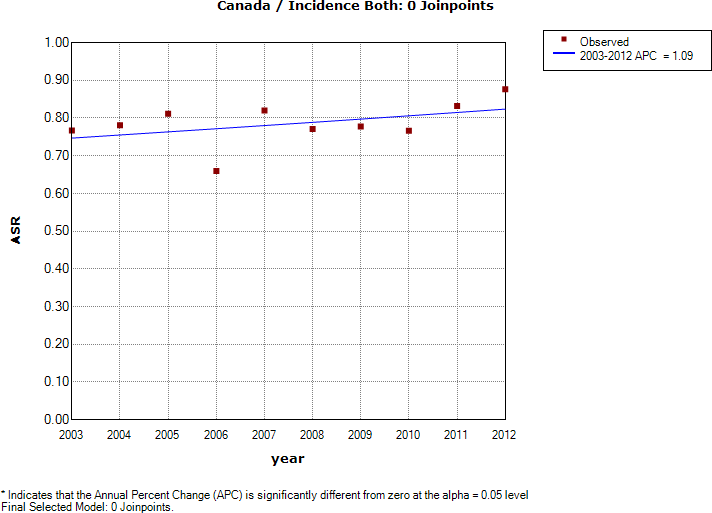 | 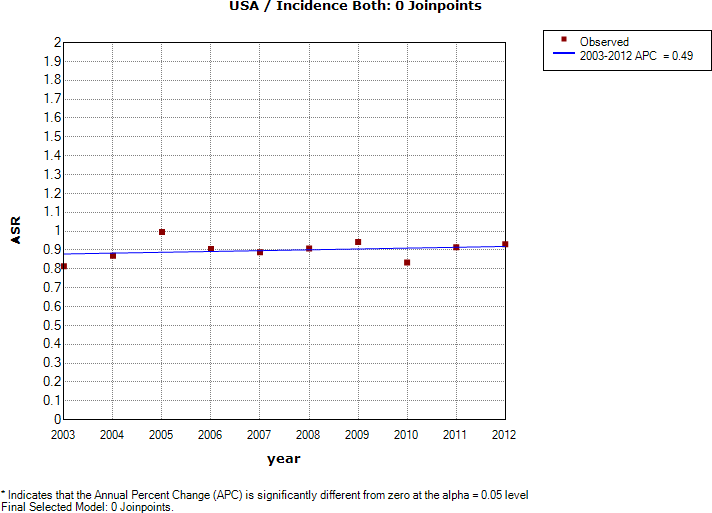 |
| **Southern America** | |
|  | 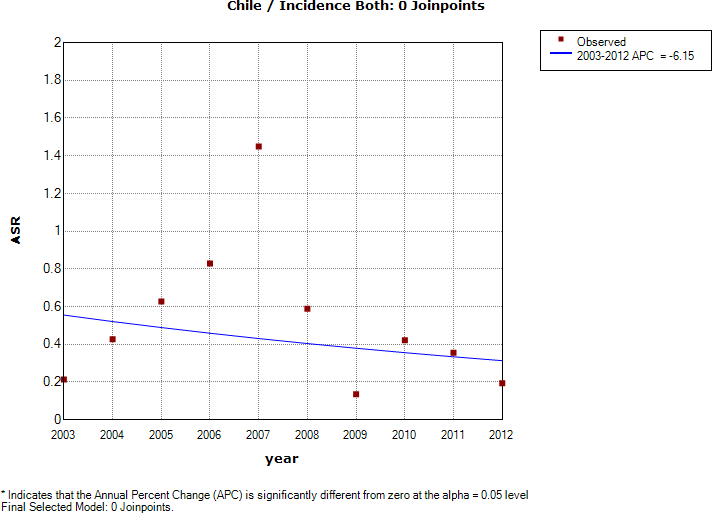 |
|  | 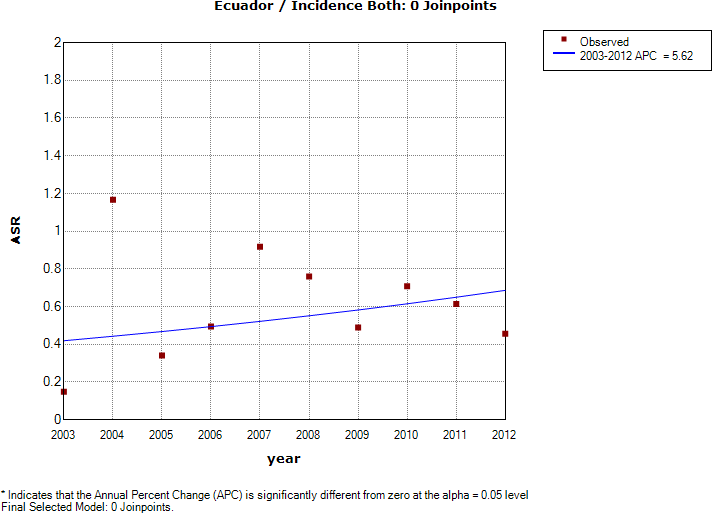 |
| 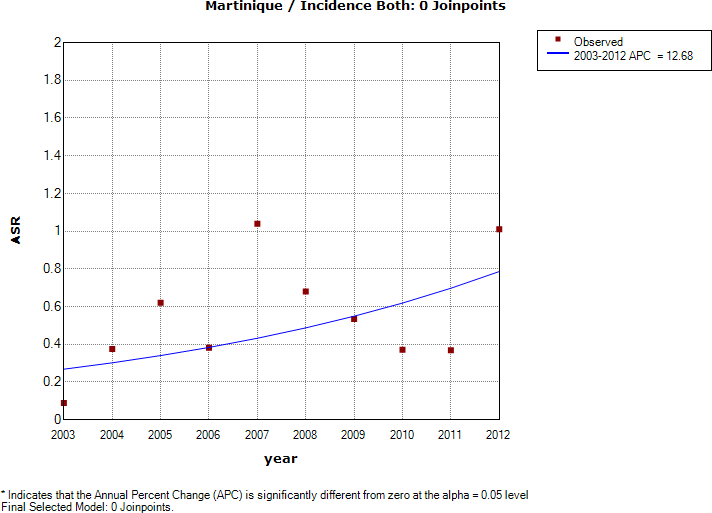 |  |


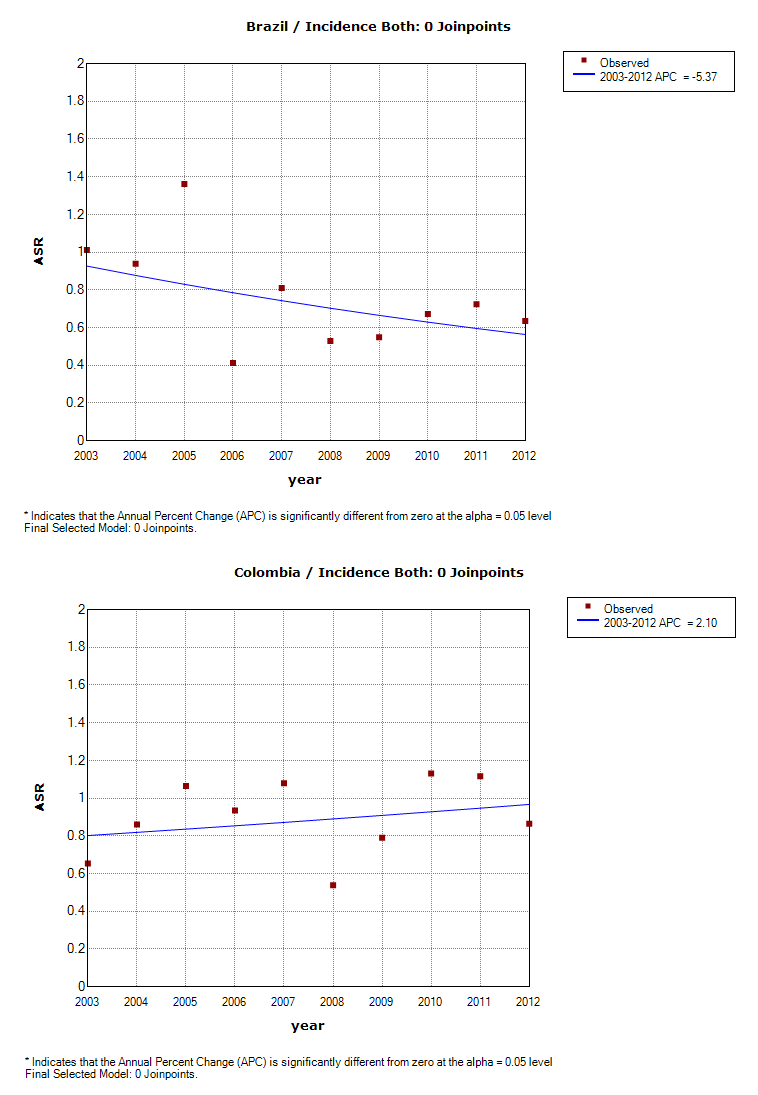


| **Northern Europe** | |
| --- | --- |
| 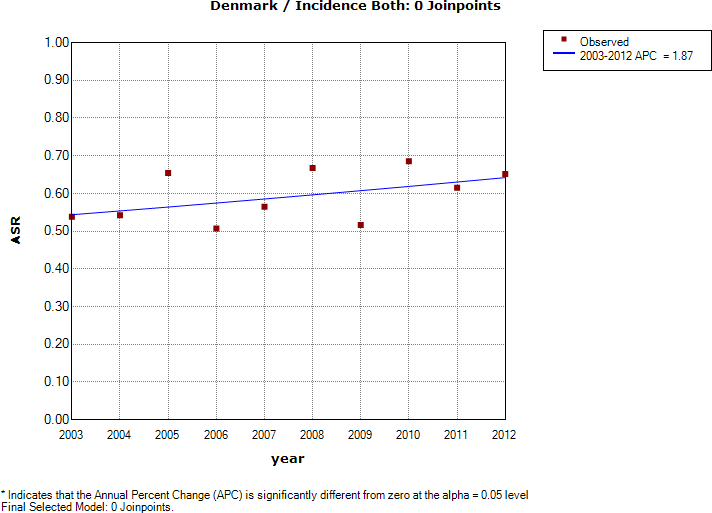 | 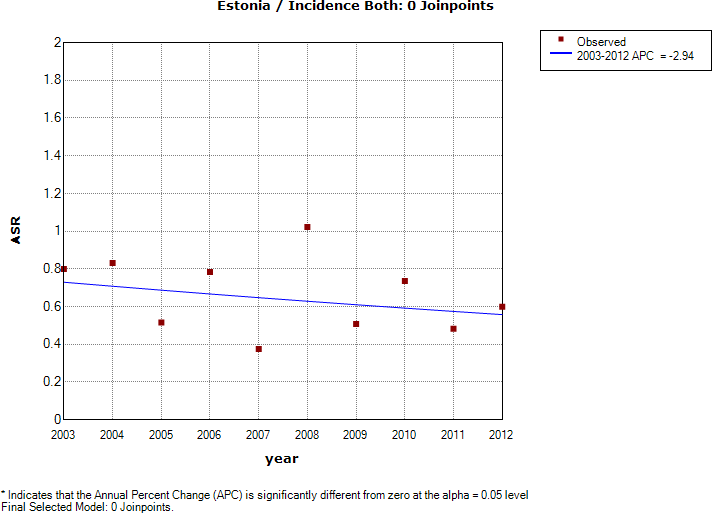 |
| 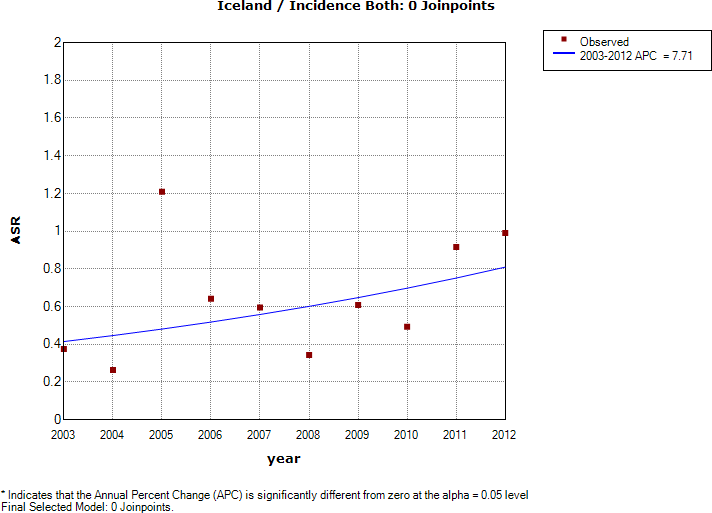 |  |
| 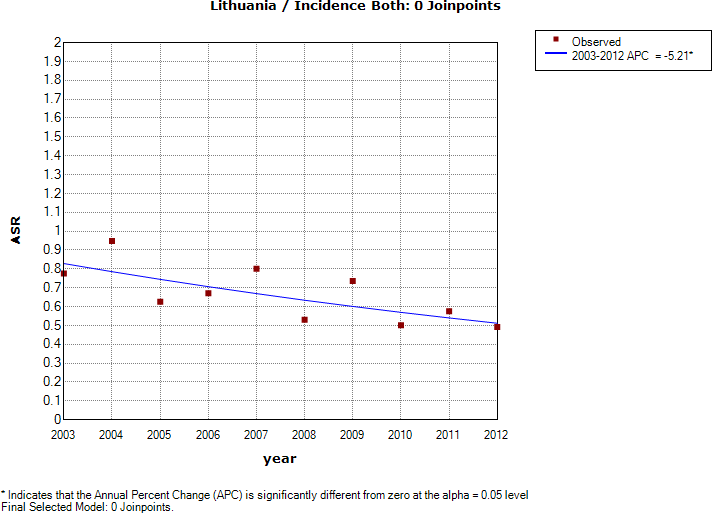 |  |
| 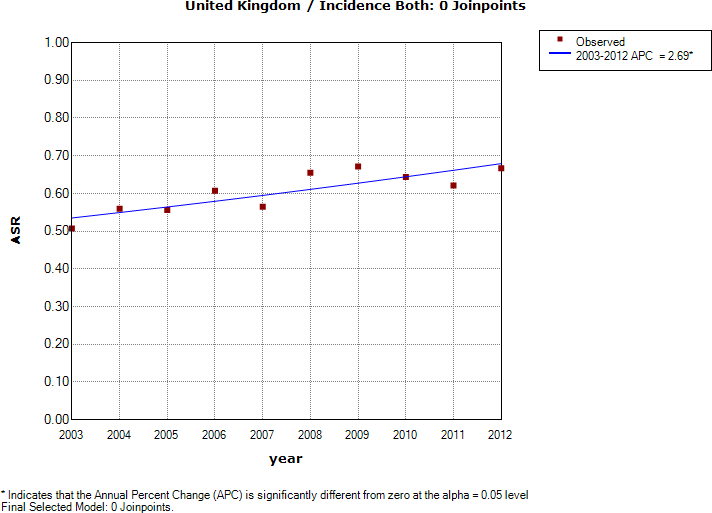 |  |


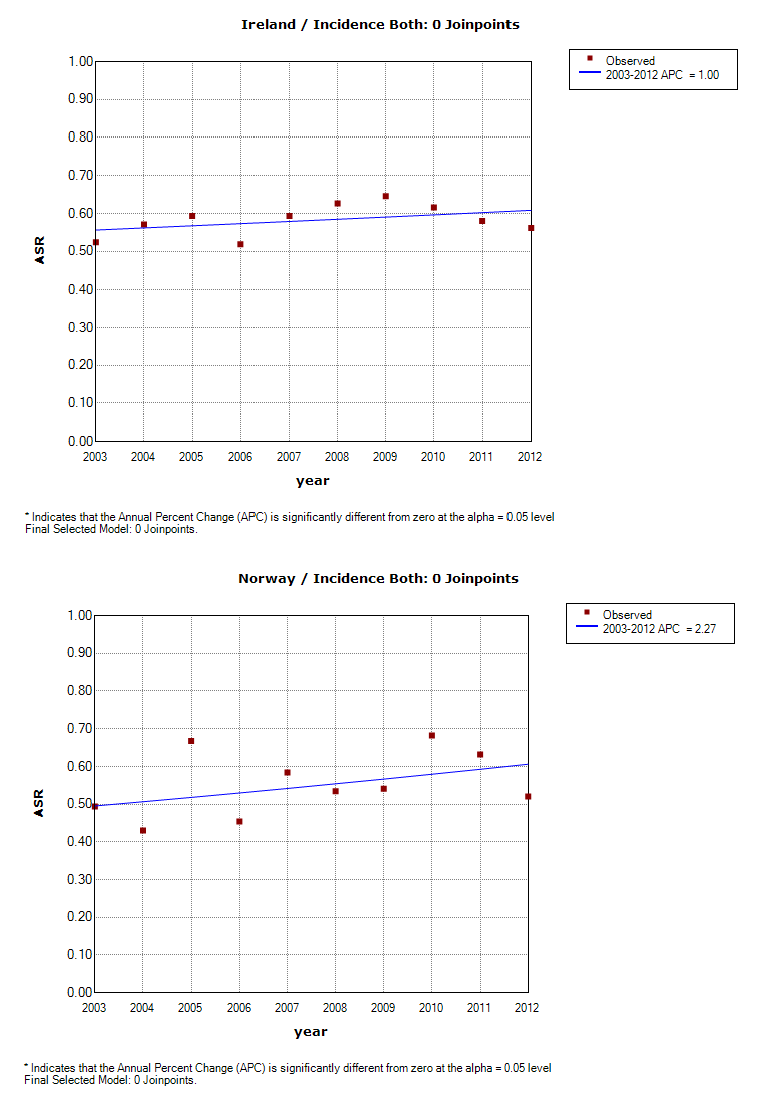


| **Western Europe** | |
| --- | --- |
| 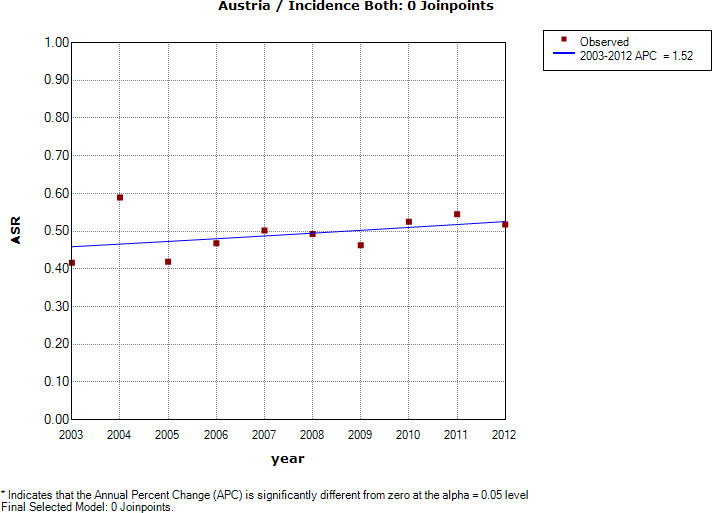 | 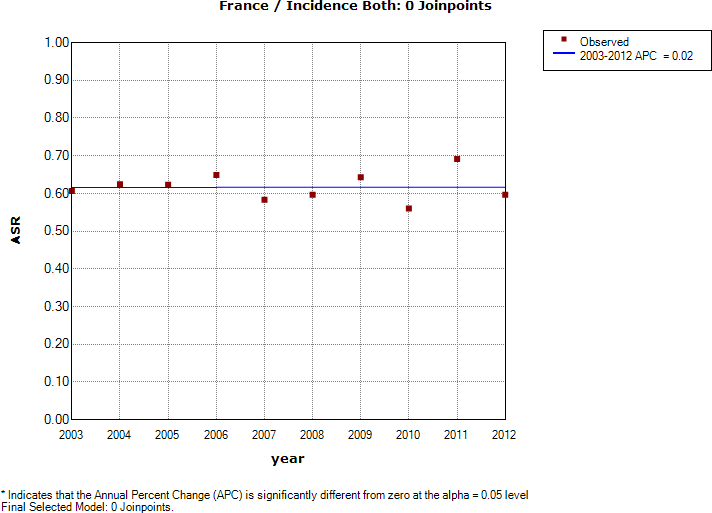 |
|  | 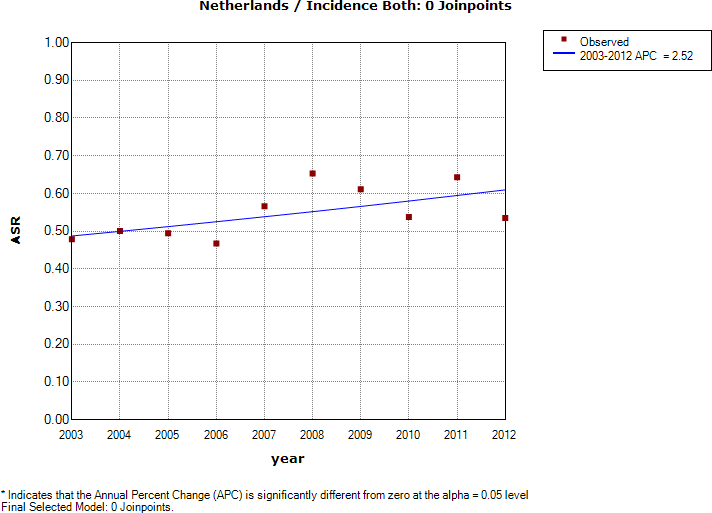 |
|  |  |


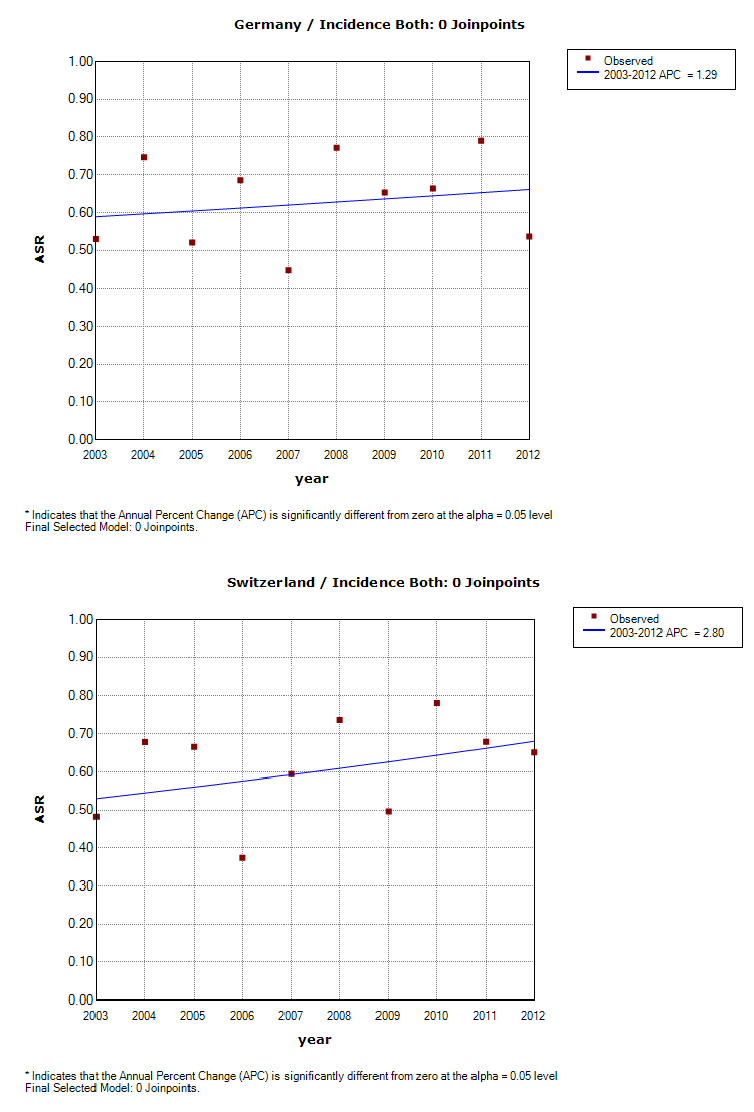


| **Southern Europe** | |
| --- | --- |
|  | 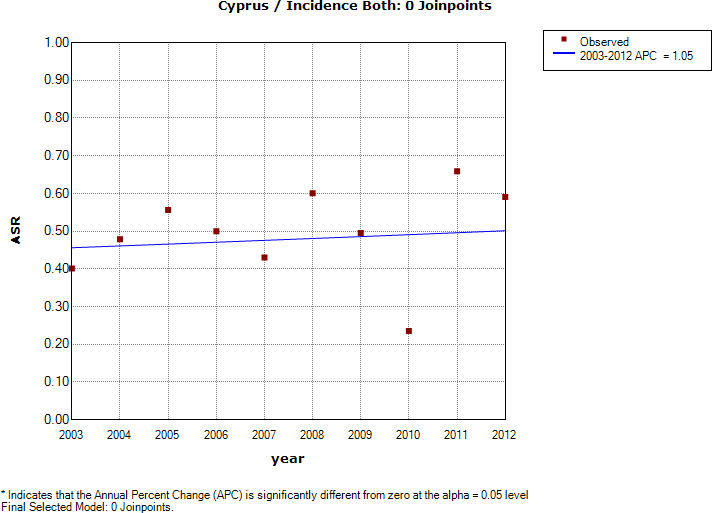 |
|  | 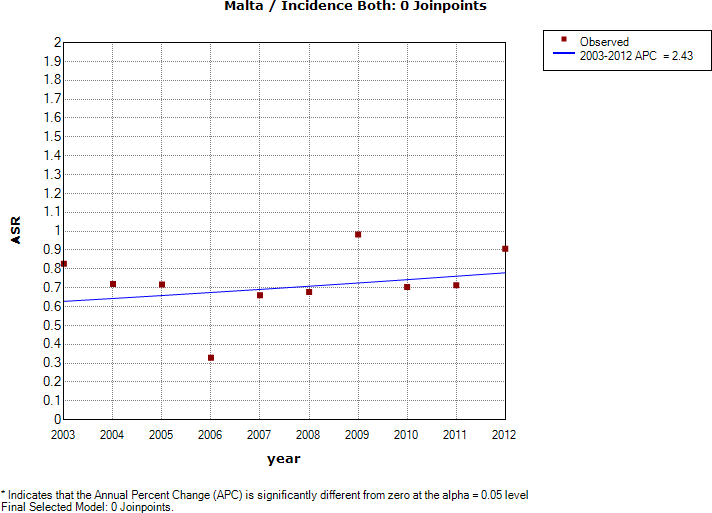 |
| 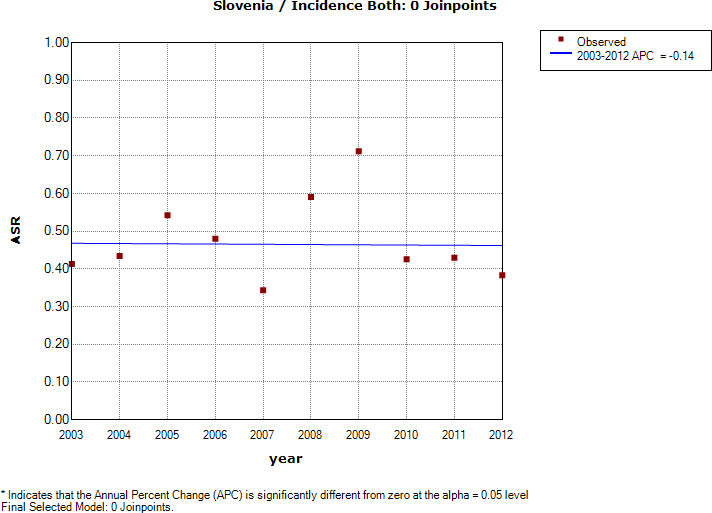 | 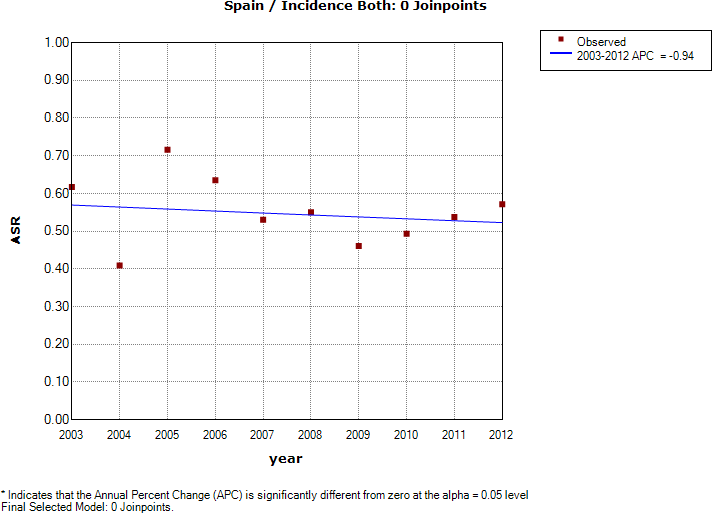 |


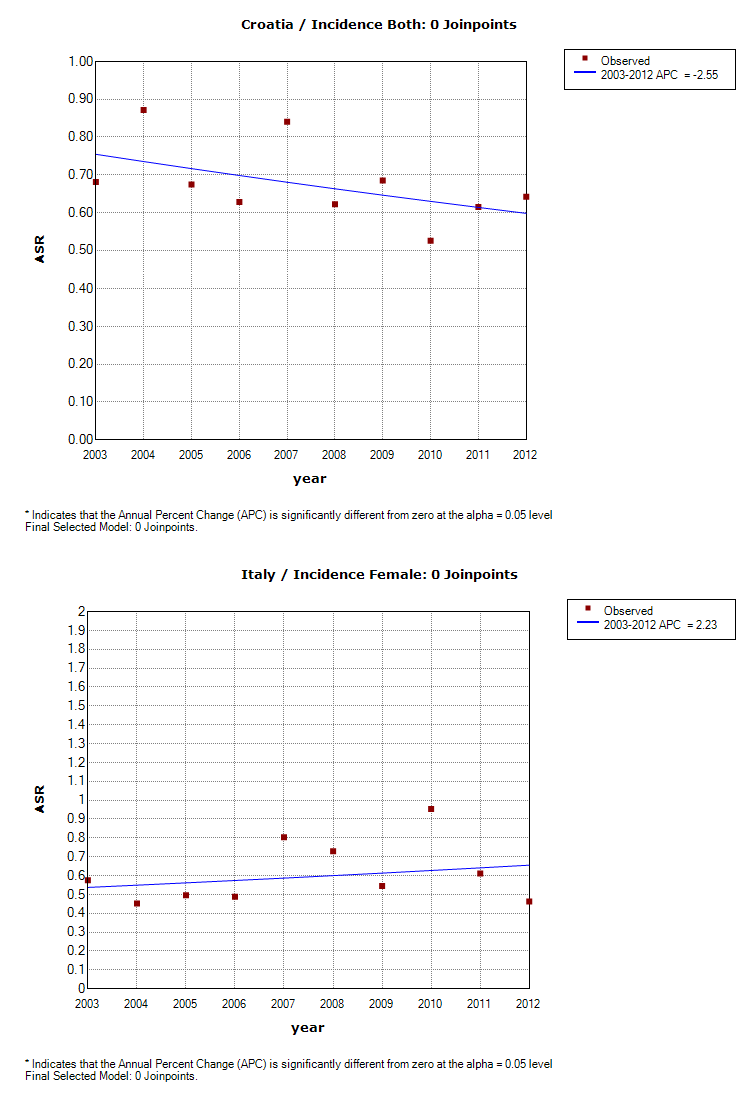


| **Eastern Europe** | |
| --- | --- |
| 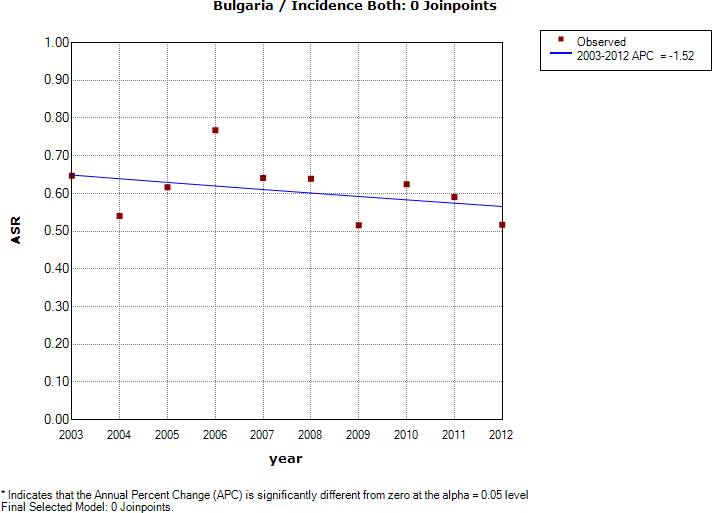 | 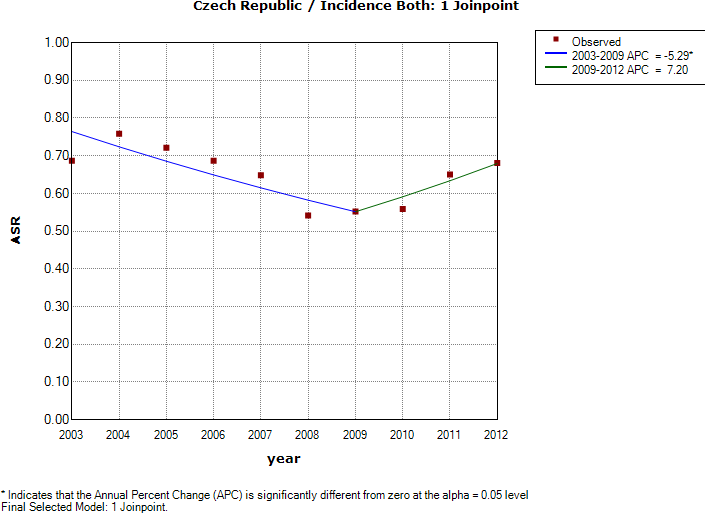 |
|  |  |
| **Africa** | |
|  |  |

d.) Young

| **Asia** | |
| --- | --- |
|  |  |
|  |  |
|  |  |

|  |  |
| --- | --- |
|  |  |
| **Oceania** | |
|  |  |

| **Northern America** | |
| --- | --- |
|  |  |
| **Southern America** | |
|  |  |
|  |  |
|  |  |

| **Northern Europe** | |
| --- | --- |
|  |  |
|  |  |
|  |  |
|  |  |

| **Western Europe** | |
| --- | --- |
|  |  |
|  |  |
|  |  |

| **Southern Europe** | |
| --- | --- |
|  |  |
|  |  |
|  |  |

| **Eastern Europe** | |
| --- | --- |
|  |  |
|  |  |
| **Africa** | |
|  |  |

### e.) Old

| **Asia** | |
| --- | --- |
|  |  |
|  |  |
|  |  |

|  |  |
| --- | --- |
|  |  |
| **Oceania** | |
|  |  |

| **Northern America** | |
| --- | --- |
|  |  |
| **Southern America** | |
|  |  |
|  |  |
|  |  |

| **Northern Europe** | |
| --- | --- |
|  |  |
|  |  |
|  |  |
|  |  |

| **Western Europe** | |
| --- | --- |
|  |  |
|  |  |
|  |  |

| **Southern Europe** | |
| --- | --- |
|  |  |
|  |  |
|  |  |

| **Eastern Europe** | |
| --- | --- |
|  |  |
|  |  |
| **Africa** | |
|  |  |
